# Single-Anchor Graph-Spectral Manifold Adaptation for Near-Field Underwater Bearing Estimation

**DOI:** 10.3390/s26216811

**Published:** 2026-10-28

**Authors:** Yi Zhang, Xiaomin Zhang, Jie Yang

**Affiliations:** 1 School of Marine Science and Technology, Northwestern Polytechnical University, Xi’an 710072, China; zhangyi61@mail.nwpu.edu.cn; 2 AVIC Shaanxi Aero Electric Co., Ltd., Xi’an 710077, China; 1521145045@mail.nwpu.edu.cn

**Keywords:** underwater acoustics, hydrophone array, near-field localization, manifold adaptation, graph signal processing, direction of arrival

## Abstract

Large-aperture hydrophone arrays at short ranges require spherical steering to represent wavefront curvature. After modeling this propagation, residual mismatch remains, and one imperfect reference observation provides limited evidence about which components will transfer reliably. We propose graph-spectral manifold adaptation for near-field bearing estimation (GSMA-NF), combining a geometry-derived spherical manifold and a physical sensor graph to constrain single-anchor residual transfer. GSMA-NF removes nominal spherical propagation from the anchor observation, represents the residual in the graph Fourier basis, and uses high-order energy as an uncertainty proxy to attenuate weakly supported low-order modes. Range-profiled multiple signal classification (MUSIC) estimates bearing with the adapted manifold, while an independent covariance rank-dominance criterion controls output release. Controlled simulations show that spherical modeling reduces short-range plane-wave error and that adaptive shrinkage limits severe negative transfer under weak-anchor conditions, while fixed graph smoothing provides comparable performance in several conditions. Model-mismatch and directional-transfer tests relate graph-prior benefit to low-order residual structure and bearing offset; interference tests delimit rank dominance to output-support assessment. Lake data show stable nominal-frame bearings across contiguous windows of a narrowband fixed-source sequence, while the screen withholds a window with insufficient rank dominance, supporting fixed-source temporal transfer in the nominal array frame.

## 1. Introduction

Hydrophone arrays support underwater direction-of-arrival (DOA) estimation for passive monitoring, bearing-only tracking, source localization, and mobile marine sensing [1,2,3,4]. This study considers a scalar pressure-sensor array; hybrid scalar–vector arrays provide an adjacent sensing architecture with additional directional measurements [5]. Reliable bearing estimation requires an array-response model that represents the deployed sensors. Subspace estimators such as multiple-signal classification (MUSIC) depend on agreement between the assumed steering response and the measured signal subspace [6,7]. For large-aperture hydrophone arrays used at short source ranges, maintaining that agreement requires both appropriate propagation modeling and control of residual channel mismatch.

**Figure 1:**
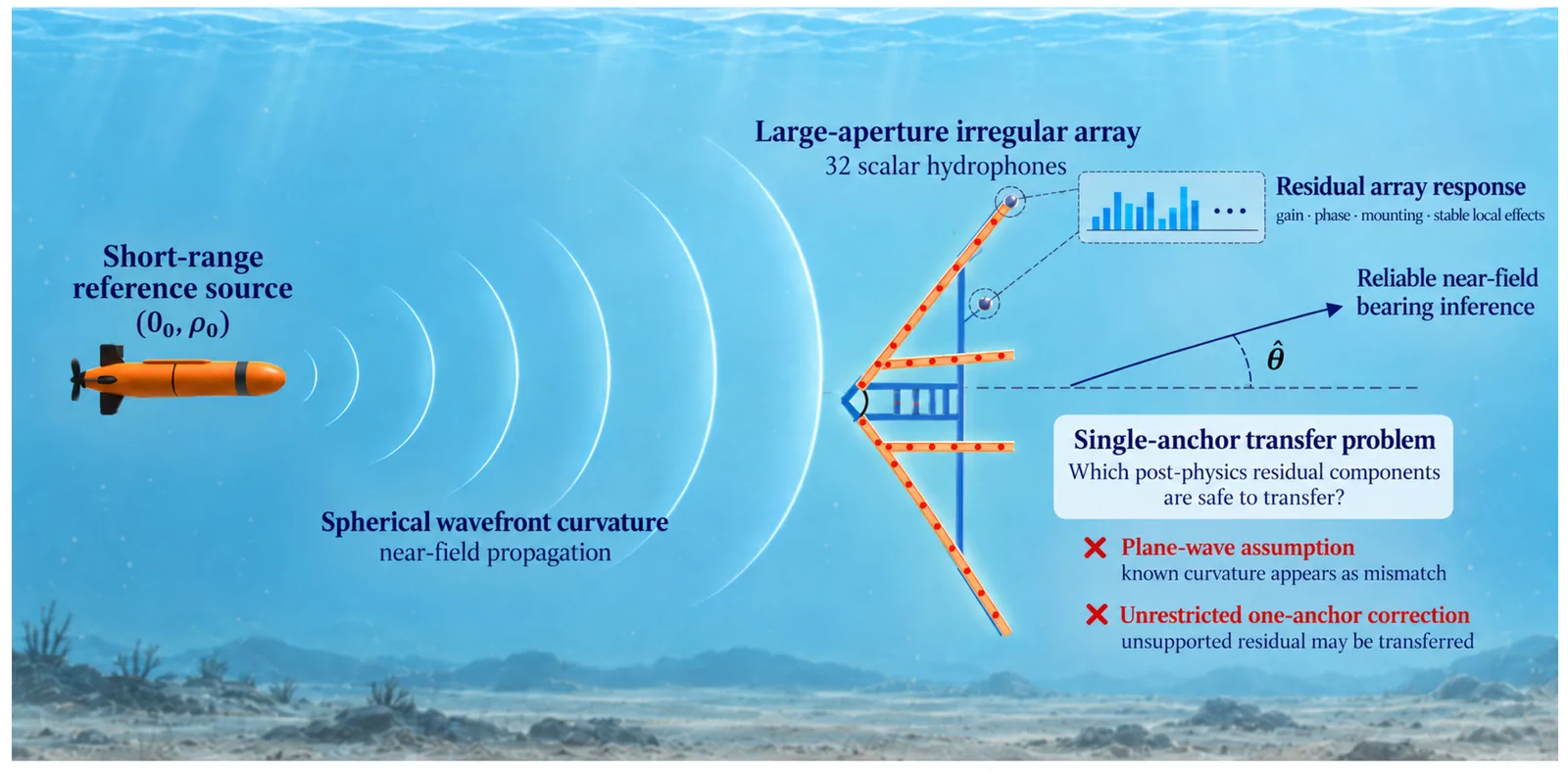
Overview of single-anchor near-field bearing estimation with spherical propagation modeling and graph-spectral residual-response adaptation.

Two coupled effects make this requirement difficult to satisfy in deployment-constrained lake and harbor measurements. A source only a few meters from a large-aperture array can lie within the Fraunhofer distance, so spherical wavefront curvature makes the steering response depend jointly on bearing and range [8,9,10]. Even after that curvature is modeled, gain and phase errors can leave a residual response mismatch in scalar and vector acoustic arrays [11,12]. Mounting and stable local propagation effects may introduce additional residual structure in field measurements. Absorbing curvature into a channel correction confounds known propagation physics with calibration. A nominal spherical manifold by itself leaves the residual mismatch untreated. When calibration is restricted to one reference emission, the additional difficulty is determining how much of the observed residual can inform subsequent measurements. Figure 1 illustrates this progression from near-field modeling to uncertain residual transfer.

Existing calibration research provides several ways to improve array-response models. Near-field acoustic methods jointly estimate source parameters and array errors using geometric constraints or diversity in source-relative observations [9,10]. More generally, self-calibration can exploit localized or cooperative sources, ambient fields, and structural constraints [13,14,15,16]. Moving ships, repeated trajectories, tow dynamics, and deformation models provide further information for recovering array shape and orientation [17,18,19,20]. Robust estimators also address sensor-position error and near-field or far-field interference on compact sonar arrays [21,22]. These methods obtain identifiability from additional observations or physical constraints. The present problem concerns which parts of a correction from one fixed reference location can be transferred when a nominal array geometry is available.

Single-source calibration is particularly relevant to this restricted setting. An eigenstructure approach uses a roughly localized source to calibrate an array of arbitrary geometry [23]. Later methods jointly estimate source direction and gain and phase errors for linear arrays [24], or exploit uniform-linear-array substructure with one source at a known direction [25]. These studies establish that one source can identify a relative array response under their respective geometric and structural assumptions. The present contribution begins with the subsequent transfer-control problem for an imperfect single observation. After compensating for the nominal spherical response, the estimated effective-response residual can still contain measurement noise, transient interference, or local multipath. Applying the complete response ratio to subsequent observations transfers these disturbances together with useful response information. Response recovery and evidence-supported manifold adaptation therefore constitute distinct objectives.

Graph signal processing offers a way to express structure on irregular sensor layouts. Early DOA methods map received array data to directed graphs and infer direction from graph-spectral structure [26,27]. Subsequent work develops narrowband graph spatial spectra and efficient estimators [28,29], including joint bearing and range estimation [30]. In these formulations, the graph acts on the observations or defines the localization spectrum. In the proposed formulation, the graph regularizes the residual response applied to a geometry-derived spherical manifold, and MUSIC supplies the bearing estimate. The graph consequently serves as a transfer prior within the localization model.

Reliability also depends on the observation processed with the adapted manifold. Recent learning-based estimators address phase-disturbed moving arrays [31] and single-snapshot underwater direction finding with limited training data [32]. Other studies consider multipath-resilient processing, uncertainty quantification, and robust sequential bearing estimation [33,34,35]. These advances highlight the need to account for limited or contaminated evidence. In the present pipeline, constrained anchor-side adaptation defines the response model, and an independent covariance screen serves as an operational safeguard for subsequent test windows.

The resulting research question concerns how to constrain the transfer of a residual inferred from one imperfect near-field reference after compensating for deterministic propagation. We address it through graph-spectral manifold adaptation for near-field bearing estimation (GSMA-NF). One reference emission and its multichannel observation constitute the anchor. Using the nominal geometry, GSMA-NF factors out the nominal spherical response and represents the remaining effective-response residual in a physical graph basis. Under the assumption that transferable residual structure is concentrated in low graph modes, the method combines a low-dimensional topology constraint with high-mode evidence that attenuates weakly supported retained modes. This uncertainty-aware residual transfer is the core methodological contribution. Range-profiled MUSIC uses the adapted response for bearing estimation, and a separate eigenvalue-based screen controls output release after manifold adaptation.

The main contributions are threefold:

(1) We formulate single-anchor near-field manifold adaptation for an irregular hydrophone array with nominal coordinates by separating geometry-derived spherical propagation from the effective response residual available for adaptation.
(2) We design an uncertainty-aware transfer rule that restricts physical-manifold modification to evidence-supported low-order graph modes.
(3) We characterize when single-anchor residual transfer remains informative through matched and model-mismatched simulations, geometry-induced directional-transfer and screening stress tests, and an operational fixed-source lake trial.

## 2. Near-Field Single-Anchor Manifold Adaptation Problem

For an irregular scalar array with nominal sensor coordinates, we consider near-field bearing estimation from one fixed reference emission with a nominal bearing and approximate range. This emission and its multichannel observation form the anchor. The model separates deterministic spherical propagation from residual channel and stable propagation mismatch, thereby restricting manifold adaptation for subsequent measurements to transferable anchor-derived information.

### 2.1. Irregular Array and Spherical Propagation

Consider *M* scalar hydrophones with nominal planar position vectors $p_{m}\in R^{2}$. Bearing θ is measured clockwise from the positive *y* axis of the nominal array coordinate system. A source at range ρ then has coordinates

(1) $$q\left(\theta ,\rho \right)=\rho {\left[\sin\theta ,\cos\theta \right]}^{T}.$$

The propagation distance to sensor *m* is $d_{m}={∥q-p_{m}∥}_{2}$. With sensor 1 as the phase reference, the spherical steering response at frequency *f* is

(2) $$a_{m}\left(\theta ,\rho ;f\right)=\frac{d_{1}}{d_{m}}\exp\left[-j\frac{2\pi f}{c}\left(d_{m}-d_{1}\right)\right],$$

where *c* is the speed of sound. The amplitude term represents geometric spreading relative to sensor 1, which serves as the array phase reference. Although this term can be omitted when phase information is the reliable component, it is retained throughout this study.

**Figure 2:**
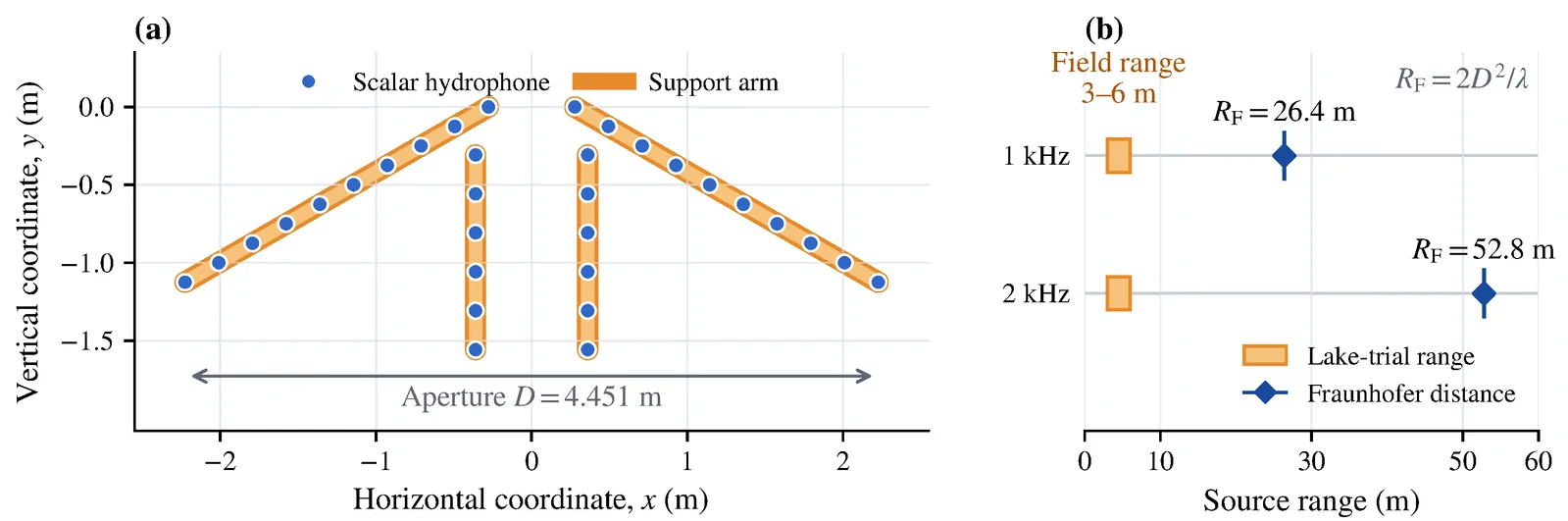
Nominal array geometry and near-field regime: (**a**) scalar-hydrophone layout; (**b**) lake-trial range and frequency-dependent Fraunhofer distances.

When ρ is much larger than the array aperture, Equation (2) approaches the plane-wave response. The transition depends on the Fraunhofer distance $R_{F}=2D^{2}/\lambda$, where *D* is the maximum aperture and λ=c/f. Figure 2a shows the nominal design coordinates used for the 32 scalar hydrophones. The irregular planar layout has a maximum aperture of D=4.451 m. Figure 2b compares the recorded lake-trial source-range interval with the Fraunhofer distances at 1 and 2 kHz; both distances substantially exceed the recorded range. The tested ranges therefore require a spherical propagation response. The complete coordinate list is provided in Supplementary Table S1, and these coordinates define the nominal processing geometry.

### 2.2. Single-Anchor Response Mismatch

For a dominant narrowband source, the measured snapshots satisfy

(3) $$x_{\ell }=s_{\ell }\mathrm{diag}\left(g_{f}\right)a\left(\theta ,\rho ;f\right)+n_{\ell },$$

where $g_{f}\in C^{M}$ is an effective response assumed to remain direction independent over the transfer considered, $s_{\ell }$ is the source coefficient, and $n_{\ell }$ contains noise and unmodeled propagation effects. In a controlled setting, $g_{f}$ represents the channel response. In a field recording, it may also absorb stable local propagation effects beyond the spherical model. The response has a common complex-scale ambiguity, which we remove using the unit-mean operator

(4) $$\mathrm{unitmean}\left(v\right)=\frac{v}{\bar{v}},\;\bar{v}=M^{-1}1^{T}v,\;\left|\bar{v}\right|>\epsilon_{m},$$

where $\epsilon_{m}=10^{-12}$ defines the numerical admissibility condition. Adaptation requires $|\bar{v}|>\epsilon_{m}$; anchors at or below this threshold are rejected because their relative-response normalization is unstable.

For any window containing $L_{w}$ snapshots, we form the sample covariance

(5) $$\hat{R}=\frac{1}{L_{w}}\sum_{\ell =1}^{L_{w}}x_{\ell }x_{\ell }^{H}.$$

The anchor and each test window are processed separately, producing ${\hat{R}}_{0}$ and ${\hat{R}}_{x}$, respectively. An eigendecomposition is applied to each covariance. Let $u_{1,f}$ denote the unit-norm principal eigenvector of ${\hat{R}}_{0}$, and let $\left(\theta_{0},\rho_{0}\right)$ denote the reference location. The raw relative correction applies Equation (4) to the propagation-compensated principal eigenvector:

(6) $${\tilde{g}}_{f}=\frac{u_{1,f}⊘a\left(\theta_{0},\rho_{0};f\right)}{M^{-1}1^{T}\left[u_{1,f}⊘a\left(\theta_{0},\rho_{0};f\right)\right]},$$

where ⊘ denotes element-wise division. Equation (6) is well defined under the admissibility condition in Equation (4). It defines the unrestricted channel-wise calibration baseline against which graph-spectral regularization is assessed.

The proposed transfer is conditional on an operational structural assumption. After the nominal spherical response is compensated, the component of the effective-response residual that persists from the anchor to later observations is assumed to be more concentrated in low graph-frequency modes than anchor-specific disturbances. High graph-frequency energy can then indicate weak support for transferring the observed correction. Transferable structure may also occur at high graph frequencies. Such energy increases the uncertainty proxy and produces a conservative adaptation that may leave part of the mismatch uncorrected.

## 3. Graph-Spectral Manifold Adaptation

### 3.1. Overview

**Figure 3:**
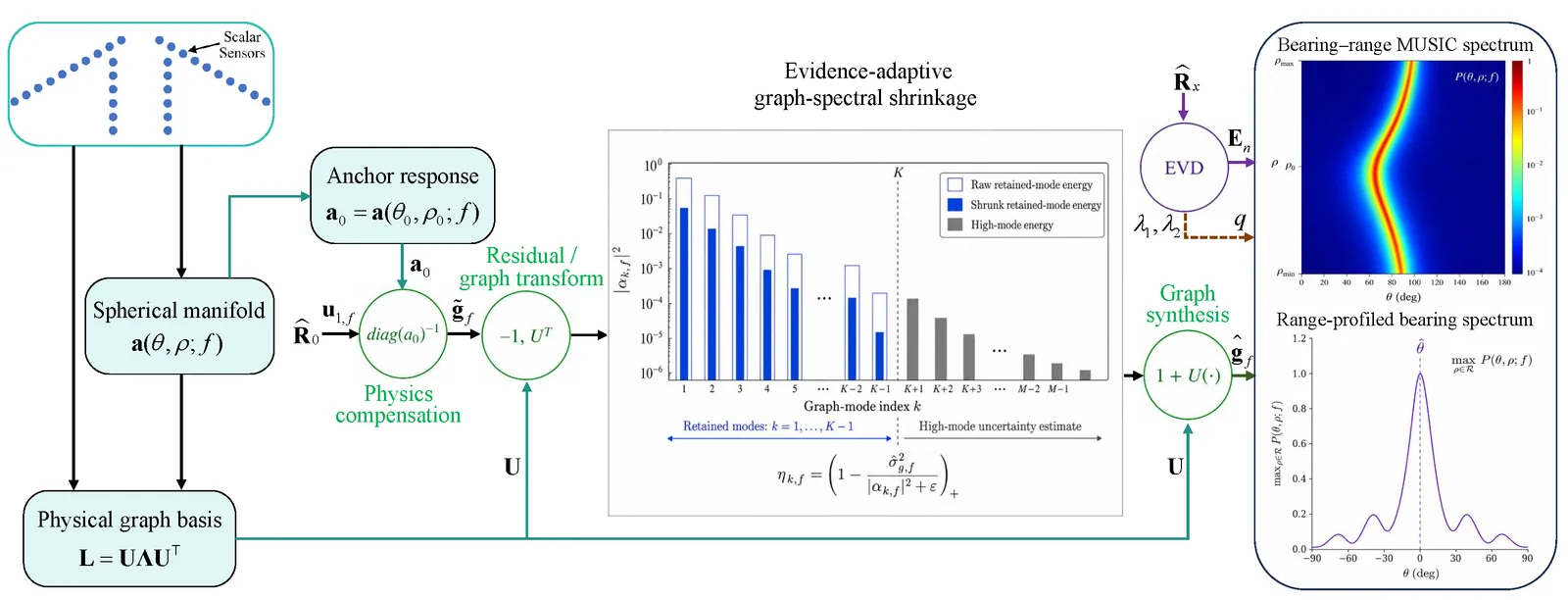
Overview of graph-spectral manifold adaptation for near-field bearing estimation (GSMA-NF), comprising anchor-based response adaptation, range-profiled multiple-signal classification (MUSIC), and rank-dominance screening.

GSMA-NF comprises a core anchor-adaptation branch followed by a downstream test branch, as summarized in Figure 3. The anchor branch uses the nominal geometry and reference location to factor out the spherical response and convert the principal covariance eigenvector into a relative effective-response ratio. A physical sensor graph decomposes this ratio into residual modes ordered from spatially smooth to oscillatory. High-mode energy provides an uncertainty proxy for the anchor, and the method transfers each low-order mode in proportion to its support in the observed evidence.

The test branch uses a separate eigendecomposition of the test covariance, combines the adapted response with spherical candidates over bearing and range, profiles the MUSIC spectrum over range, and evaluates leading-component dominance independently of the candidate bearing. The components have a deliberate hierarchy: propagation modeling provides the physical manifold, graph-spectral shrinkage controls residual transfer, MUSIC performs bearing estimation, and rank-dominance screening provides an operational output criterion that remains independent of anchor adaptation.

### 3.2. Graph Representation of the Residual Response

We represent the array as a weighted undirected graph, a standard model for signals supported on an irregular physical domain [36,37]. The edge weights are

(7) $$w_{mn}=\left\{\begin{array}{cc} \exp[-{∥p_{m}-p_{n}∥}_{2}^{2}/h^{2}], & {∥p_{m}-p_{n}∥}_{2}\leq r_{g},\\ 0, & \mathrm{otherwise}, \end{array}\right.$$

where *h* is the spatial bandwidth and $r_{g}=1.05h$ is the nominal radius. We augment this radius graph with the three nearest-neighbor links of every sensor. Each added link uses the same Gaussian weight, the union of the two edge sets is symmetrized by retaining the larger directed weight, and self-links are removed. This deterministic construction yields a connected graph for the nominal geometry. With $D_{g}=\mathrm{diag}\left(W1\right)$, the graph Laplacian is $L=D_{g}-W=U\Lambda U^{T}$. Its eigenpairs are indexed such that $0=\xi_{0}<\xi_{1}\leq \cdots \leq \xi_{M-1}$ and $u_{0}\propto 1$. The eigenvectors form a graph Fourier basis ordered from spatially smooth to oscillatory modes. The distinct notation $\xi_{k}$ and $\lambda_{i}$ is used for graph-Laplacian and test-covariance eigenvalues, respectively.

Define the mean-free anchor residual as $z_{f}={\tilde{g}}_{f}-1$ and its graph coefficients as $\alpha_{f}=U^{T}z_{f}$. Mean removal suppresses the direct-current mode $u_{0}$. The modes indexed by k=1,…,K−1 span the retained nonconstant subspace. We adopt the approximate perturbation model $\alpha_{k,f}=\beta_{k,f}+\nu_{k,f}$, where $\beta_{k,f}$ denotes transferable structure and $\nu_{k,f}$ denotes anchor-specific disturbance. For k≥K, the high modes are treated as disturbance-dominated under the operational assumption stated in Section 2, with circular complex Gaussian coefficients of mode-independent variance. Under this working approximation, $|\nu_{k,f}{|}^{2}$ is exponentially distributed with median $\sigma_{g,f}^{2}\ln2$. Perturbations introduced by the eigenvector ratio and unit-mean normalization can vary across sensors and modes. We therefore use the resulting statistic as an empirical shrinkage-control quantity. The high-order coefficients yield the robust, data-derived uncertainty proxy

(8) $${\hat{\sigma }}_{g,f}^{2}=\frac{{\mathrm{median}}_{k\geq K}{\left|\alpha_{k,f}\right|}^{2}}{\ln2}.$$

The retained coefficients are shrunk by

(9) $$\eta_{k,f}={\left(1-\frac{{\hat{\sigma }}_{g,f}^{2}}{\max(|\alpha_{k,f}{|}^{2},\epsilon )}\right)}_{+},\;1\leq k<K,$$

where ${\left(v\right)}_{+}=\max\left(v,0\right)$ and $\epsilon =10^{-12}$ prevents division by zero after unit-mean response normalization. Transferable energy above the cutoff also increases ${\hat{\sigma }}_{g,f}^{2}$ and strengthens the contraction, which can under-adapt the residual response when transferable structure extends beyond the retained subspace. The adapted effective response is

(10) $${\hat{g}}_{f}=1+\sum_{k=1}^{K-1}\eta_{k,f}\alpha_{k,f}u_{k},$$

followed by the normalization in Equation (4). This empirical Wiener-type rule uses spectral energy as a shrinkage proxy for eigenvector-ratio uncertainty. When the anchor is clean, Equation (9) approaches the raw low-dimensional correction. As unsupported high-mode energy increases, the estimate contracts toward the physical manifold. Specifically, because $0\leq \eta_{k,f}\leq 1$ and U is orthonormal, the retained raw residual bounds the norm of the adapted residual before the final common-scale normalization. Increasing ${\hat{\sigma }}_{g,f}^{2}$ monotonically reduces each active correction.

To isolate evidence-adaptive shrinkage from dimensionality reduction, an unshrunk low-order comparator uses the same basis and cutoff as GSMA-NF but sets every retained weight to one:

(11) $${\hat{g}}_{f,\mathrm{low}}=1+\sum_{k=1}^{K-1}\alpha_{k,f}u_{k},$$

followed by unit-mean normalization. Thus, Equations (11) and (10) differ only through the evidence weights $\eta_{k,f}$. A second structural comparator uses the same physical Laplacian with fixed graph smoothing:

(12) $${\hat{g}}_{f,\mathrm{fixed}}={\left(I+\mu L\right)}^{-1}{\tilde{g}}_{f},$$

followed by unit-mean normalization. We set μ=2 in all reported experiments. This comparator distinguishes the effect of a fixed graph-smoothness prior from that of the evidence-adaptive shrinkage in Equations (9) and (10).

### 3.3. Bearing Estimation and Output Screening

For a test covariance ${\hat{R}}_{x}$, let $E_{n}$ denote the noise subspace under the single-source model. Because Equation (2) retains relative geometric spreading, the candidate norm varies with bearing and range. We therefore use a unit-norm adapted candidate and the corresponding scale-invariant MUSIC spectrum

(13) $$\bar{a}=\frac{\mathrm{diag}\left({\hat{g}}_{f}\right)a\left(\theta ,\rho ;f\right)}{{∥\mathrm{diag}\left({\hat{g}}_{f}\right)a\left(\theta ,\rho ;f\right)∥}_{2}},\;P\left(\theta ,\rho ;f\right)={\left[{\bar{a}}^{H}E_{n}E_{n}^{H}\bar{a}\right]}^{-1}.$$

This normalization removes the common candidate scale and preserves sensor-to-sensor amplitude ratios and spherical phase curvature. We treat range as a nuisance variable and estimate the bearing as

(14) $$\hat{\theta }=\arg\max_{\theta }\max_{\rho \in R}P\left(\theta ,\rho ;f\right),$$

where R is a prescribed engineering range interval. All subspace baselines, including the far-field, spherical, calibrated, and response-oracle methods, follow the same unit-norm candidate convention.

Compatibility with the dominant-source assumption is screened using

(15) $$q=\frac{\lambda_{1}}{\lambda_{1}+\lambda_{2}},$$

where $\lambda_{1}\geq \lambda_{2}$ are the two largest eigenvalues of the test covariance. A positive eigensum $\lambda_{1}+\lambda_{2}$ is required to evaluate Equation (15). For a nonzero positive semidefinite covariance, q∈[0.5,1] measures the dominance of the leading covariance component, and the system releases the bearing when $q\geq q_{\min}$. The rule depends exclusively on the two leading covariance eigenvalues and serves as a rank-dominance heuristic for the adopted observation model. Coherent multipath, model mismatch, or a dominant interferer may also produce a high score. The threshold therefore selects an empirical operating point between output coverage and accepted-window error under the tested observation model.

### 3.4. Computational Requirements

Let G=|Θ||R| denote the number of bearing–range candidates. For a fixed nominal array, forming the pairwise graph and computing its eigensystem require $O(M^{2})$ and $O(M^{3})$ operations, respectively, and are performed once. The spherical candidate library requires O(MG) operations per frequency and can also be precomputed. For an anchor with $L_{0}$ snapshots, covariance formation and eigendecomposition require $O(M^{2}L_{0}+M^{3})$ operations. The full graph transform used to estimate the high-mode uncertainty adds $O(M^{2})$ operations. Shrinkage and reconstruction add at most O(MK) once the coefficients are available.

For each test window with *L* snapshots, direct range-profiled MUSIC requires $O(M^{2}L+M^{3}+GM^{2})$ operations when the full noise-subspace projection in Equation (13) is evaluated for every candidate. Its memory requirement is $O(MG+M^{2})$ for the candidate library and covariance-scale matrices. GSMA-NF changes the candidate library through a diagonal response update, performs graph adaptation once per anchor, and reuses the adapted library across test windows. It retains the asymptotic per-window order of spherical MUSIC; the bearing–range scan remains the dominant term when *G* is large.

## 4. Experiments

### 4.1. Experimental Setup and Metrics

All simulations use the nominal 32-element scalar-array geometry in Figure 2. Target bearings are drawn independently from a uniform distribution over $\left[0^{\circ },360^{\circ }\right)$, except in the controlled angular-transfer study. Source coefficients and sensor noise are independent zero-mean circular complex Gaussian variables. For each trial, the noise variance is scaled relative to the mean sensor-domain signal power to obtain the prescribed signal-to-noise ratio (SNR). Methods compared within a condition use identical response, bearing, source, and noise realizations. The propagation and graph-parameter sweeps use 200 paired trials per condition. Trial counts for the other studies are stated where those studies are introduced and summarized in Supplementary Table S2. Each experiment group uses a distinct fixed pseudorandom stream. The computational workflow used Python 3.13.5, NumPy 2.1.3, SciPy 1.15.3, and Matplotlib 3.10.0.

The numerical studies use a sound speed of 1500 m/s and a nominal anchor at $226^{\circ }$ and 4.5 m. Unless varied in a study, the anchor has 0 dB SNR and 20 snapshots, the graph bandwidth is h=0.55 m, the mode cutoff is K=8, and the output threshold is $q_{\min}=0.8$.

The primary ablations generate direction-independent channel responses from one constant and seven nonconstant graph modes. Independent Gaussian graph coefficients are scaled to produce channel-gain and phase standard deviations of 3 dB and $35^{\circ }$, respectively. The seven nonconstant modes coincide with the nominal cutoff and define a deliberately matched low-dimensional condition. This condition isolates the effect of anchor uncertainty when the transferability assumption is satisfied. The separate Gaussian-process stress test relaxes the exact graph-mode construction and varies the concentration of the residual across the retained and excluded graph modes. It therefore tests the structural assumption directly before drawing independent zero-mean gain and phase fields from the spatial covariance

(16) $$C_{mn}\left(\ell \right)=\exp\left(-\frac{{∥p_{m}-p_{n}∥}_{2}^{2}}{2\ell^{2}}\right),$$

where *ℓ* is the residual correlation length. Let b and ϕ denote the sampled gain field in decibels and phase field in radians, respectively. Each realization is rescaled to gain and phase standard deviations of 3 dB and $35^{\circ }$, respectively, and converted to the unit-mean response $g=\mathrm{unitmean}[10^{b/20}⊙\exp\left(j\phi \right)]$. This stress test uses 2 kHz, a 5 m target at 0 dB with 30 snapshots, and a −10 dB anchor with eight snapshots.

The numerical evaluation fixes the information set to the nominal irregular geometry, one reference location, and identical anchor and test observations across the compared methods. Within this protocol, the comparison spans far-field MUSIC, uncalibrated spherical MUSIC, raw-anchor calibration, unshrunk low-order projection, fixed-graph smoothing, topology-free reliability shrinkage, GSMA-NF, and a response oracle. Published single-source methods for linear or uniform linear arrays derive identifiability from those array structures [24,25], whereas joint near-field methods use additional source-relative observations [9,10]. These methods define adjacent problem settings and provide literature context. The numerical baselines form a controlled comparison under the same information set. In nominal exact-geometry simulations, the response oracle uses the exact direction-independent response, so its remaining error arises from target noise, finite snapshots, and search discretization. In the sensor-position-error and direction-dependent transfer studies, it retains the nominal processing geometry and therefore also retains geometry mismatch. The unshrunk projection isolates adaptive shrinkage from the shared low-order graph constraint. The Gaussian-process stress test also includes a topology-free reliability-shrinkage baseline,

(17) $$\hat{g}=1+\gamma \left(\tilde{g}-1\right),\;\gamma =\frac{L10^{\mathrm{SNR}/10}}{1+L10^{\mathrm{SNR}/10}},$$

where *L* is the prescribed number of anchor snapshots and the prescribed anchor SNR is expressed in decibels. This non-graph comparator receives the prescribed anchor reliability directly. GSMA-NF estimates its shrinkage proxy from the observation.

The primary error is the circular absolute error

(18) $$e_{i}=\left|{\mathrm{wrap}}_{\left[-180,180\right)}\left({\hat{\theta }}_{i}-\theta_{i}\right)\right|.$$

Depending on the function of each simulation, we report the mean absolute error (MAE), empirical cumulative distribution function, median error, 90th- or 95th-percentile error, and the probability of an error greater than $5^{\circ }$. Method contrasts use trial-wise paired differences. Mean contrasts use percentile bootstrap intervals, and binomial output and gross-error probabilities use Wilson intervals. The 200-trial mean-error sweeps use normal-approximation intervals. The primary range sweep uses on-grid source ranges to isolate propagation and response effects. A separate study samples the true range continuously and varies bearing-grid spacing, range-grid spacing, and search boundaries. Field results are reported as nominal-coordinate descriptive statistics, while truth-referenced error metrics are confined to the controlled simulations.

### 4.2. Near-Field Model and Adaptation Effects

**Figure 4:**
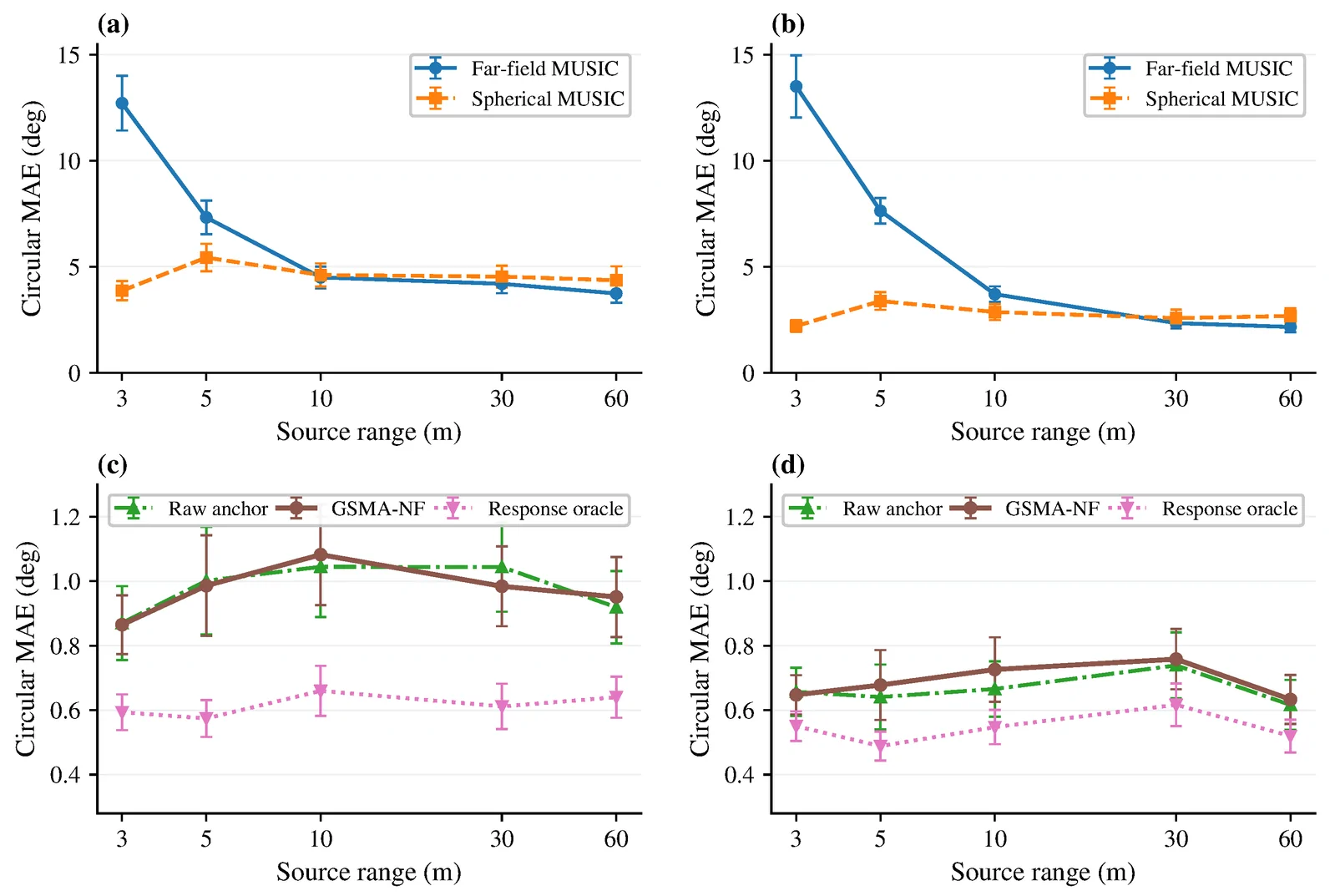
Mean absolute circular bearing error (MAE) versus source range for (**a**,**b**) propagation-model and (**c**,**d**) anchor-adaptation comparisons; error bars denote 95% normal-approximation confidence intervals.

The propagation-model study uses 30 target snapshots at 0 dB and evaluates 1 and 2 kHz across true ranges of {3,5,10,30,60} m; the same five ranges form the candidate set R. In Figure 4a,b, plane-wave processing exceeds $12^{\circ }$ error at 3 m, whereas spherical processing remains within a few degrees and approaches the far-field result as the range increases. Figure 4c,d place the response-adapted methods on a much smaller error scale: raw-anchor calibration and GSMA-NF remain near $1^{\circ }$ and follow the response-oracle trend across the sampled ranges. The confidence intervals describe sampling uncertainty, and the on-grid range search isolates propagation and response effects from range-discretization error.

### 4.3. Anchor-Quality and Structural Ablation

**Figure 5:**
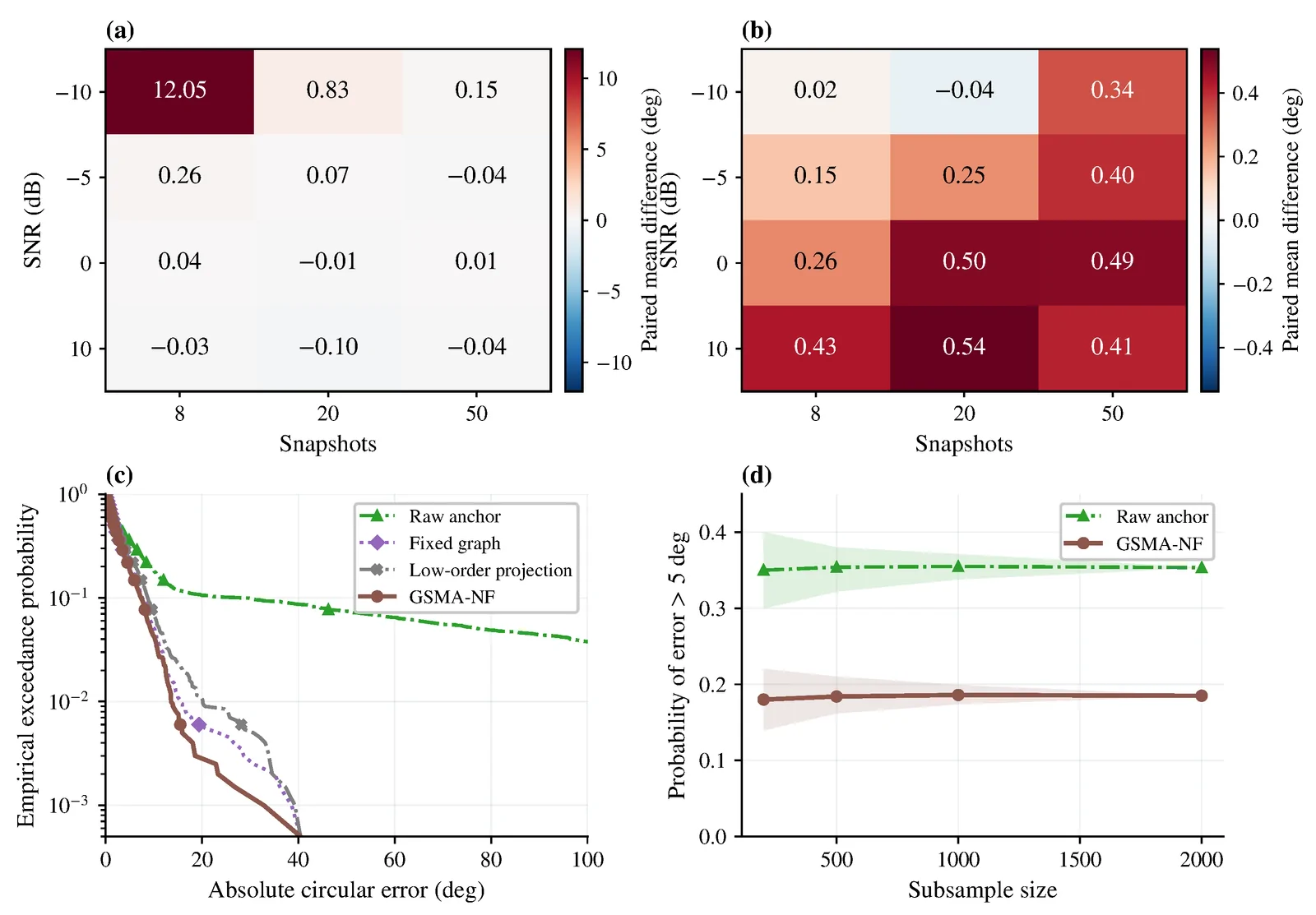
Anchor-quality and heavy-tail analysis: paired mean differences for (**a**) raw transfer and (**b**) fixed-graph smoothing relative to GSMA-NF across anchor signal-to-noise ratio (SNR) and snapshot count, (**c**) weak-anchor exceedance distributions, and (**d**) gross-error stability under repeated subsampling.

At 2 kHz with a 5 m target at 0 dB and 30 snapshots, the anchor-quality study varies anchor SNR over {−10,−5,0,10} dB and anchor snapshots over {8,20,50}, using 500 paired trials per condition. Figure 5a,b compare raw transfer, fixed-graph smoothing, unshrunk projection, and GSMA-NF on these paired observations. The largest raw-transfer penalty occurs at −10 dB anchor SNR with eight snapshots, where the paired mean raw-minus-GSMA-NF difference is $12.05^{\circ }$. This separation approaches zero and occasionally changes sign as anchor support increases. Fixed-graph smoothing remains a strong comparator. Its paired mean difference from GSMA-NF ranges from $-0.04^{\circ }$ to $0.54^{\circ }$ across the anchor grid, which is much smaller than the raw-transfer difference.

The dedicated 2000-trial weak-anchor study in Figure 5c,d evaluates the adverse-error tail at −10 dB anchor SNR and eight anchor snapshots. The median errors are $2.57^{\circ }$ for raw transfer and $1.75^{\circ }$ for GSMA-NF. Raw transfer has a 95th-percentile error of $77.27^{\circ }$ and a gross-error probability of 0.354 with a 95% Wilson interval of [0.333, 0.375]. The corresponding GSMA-NF values are $9.33^{\circ }$ and 0.185 [0.169, 0.203]. Fixed-graph smoothing gives a 95th percentile of $10.10^{\circ }$ and a gross-error probability of 0.206. The paired mean fixed-minus-GSMA-NF difference is $0.20^{\circ }$ with a 95% bootstrap interval of [$0.05^{\circ }$, $0.34^{\circ }$]. Unshrunk projection minus GSMA-NF is $0.63^{\circ }$ [$0.46^{\circ }$, $0.81^{\circ }$]. Repeated subsampling shows that the raw-transfer tail is much less stable at 200 trials. The separation in gross-error probability persists as the sample size increases. These results support evidence-adaptive control of unsupported transfer within the tested regime; fixed smoothing remains competitive in several conditions.

**Figure 6:**
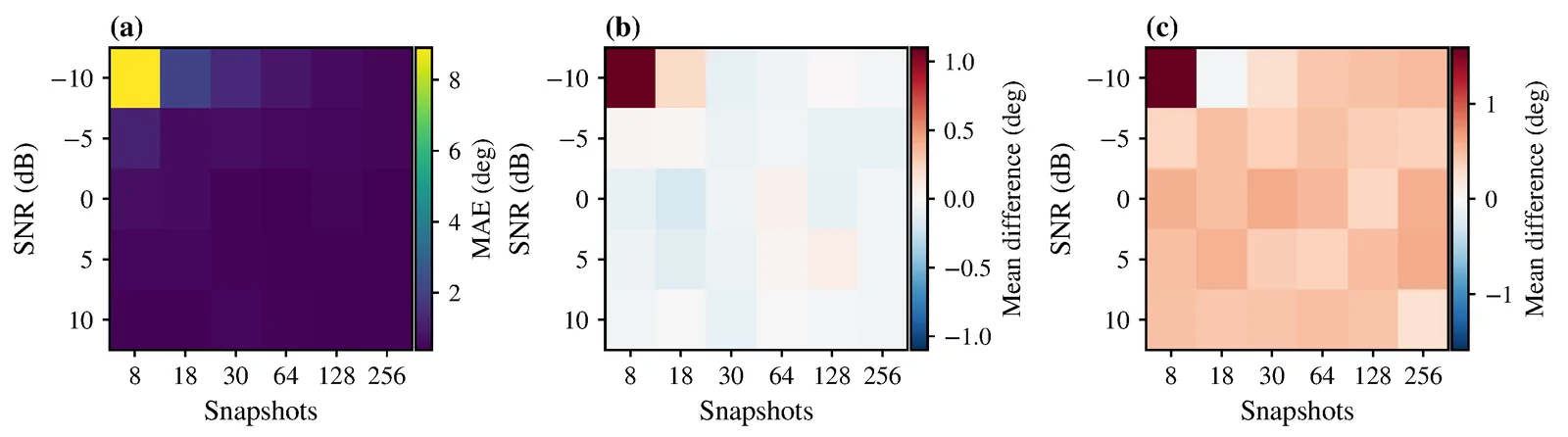
Target-side support analysis across SNR and snapshot count: (**a**) GSMA-NF error, (**b**) raw-transfer difference, and (**c**) fixed-graph difference relative to GSMA-NF.

At 2 kHz and a 5 m target range, the target-support study varies target SNR over {−10,−5,0,5,10} dB and target snapshots over {8,18,30,64,128,256}, using 300 paired trials per condition and the nominal 0 dB, 20-snapshot anchor. The three heatmaps in Figure 6 separate absolute GSMA-NF error from paired method differences. Figure 6a concentrates the largest error at −10 dB with eight snapshots and places the field-like count of 18 snapshots at 0 dB in the low-error region. In Figure 6b, the raw-minus-GSMA-NF advantage is largest in the weakest-support cell and otherwise remains close to zero, with values from $-0.18^{\circ }$ to $1.10^{\circ }$. The predominantly positive fixed-minus-GSMA-NF differences in Figure 6c are modest across the grid. These patterns localize the value of adaptive shrinkage to weak target support while showing little separation from the transfer baselines when the target observation is well supported.

The parameter sweep is a local numerical-sensitivity analysis around the nominal design and reuses the same paired observations. It uses the matched residual generator at 2 kHz with a 5 m target, 0 dB target and anchor SNRs, 30 target snapshots, and 20 anchor snapshots. Table 1 indicates little sensitivity to graph bandwidth over 0.45–0.65 m. Using four modes underfits the matched generator. Values of K≥8 define a stable region with MAE values between $0.63^{\circ }$ and $0.76^{\circ }$. The nominal choice K=8 is the smallest tested value on this plateau. The sweep therefore characterizes numerical sensitivity under the matched residual generator.

**Figure 7:**
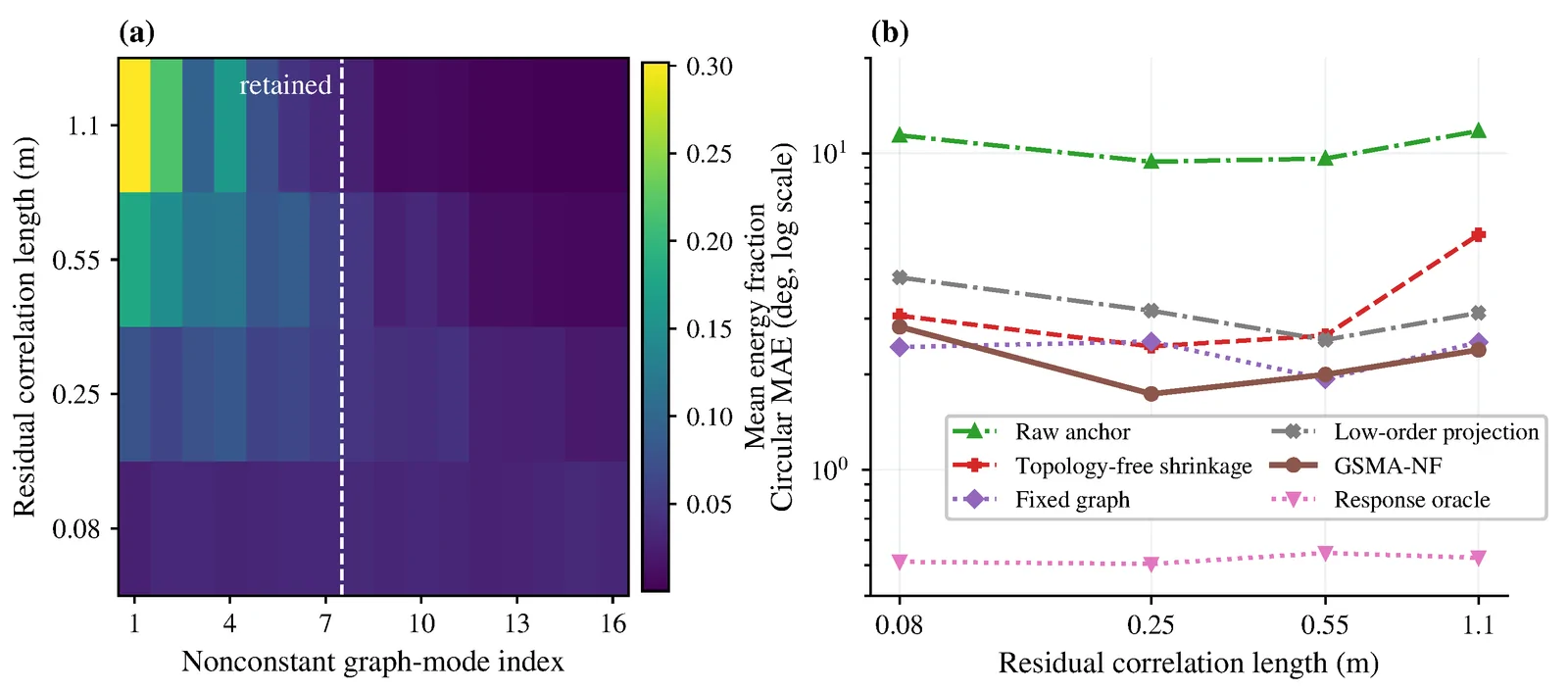
Gaussian-process residual-mismatch analysis: (**a**) residual-energy distribution across graph modes; (**b**) bearing-error sensitivity to spatial correlation length.

The matched ablation conflates the effect of topology with the generic effect of reducing calibration freedom. The independent Euclidean Gaussian-process stress test in Figure 7 draws residuals directly from a spatial covariance. Figure 7a reveals a progressively stronger concentration in the retained low-order graph modes as the correlation length increases; the shortest-scale residual is visibly the least compatible with the transferability assumption. Consistent with that change, Figure 7b places raw calibration at the highest point estimate throughout and makes graph regularization more competitive for smoother residuals. Table 2 reports paired mean circular-error differences with 95% percentile bootstrap intervals, where positive values favor GSMA-NF. Low-order projection minus GSMA-NF is positive at every sampled correlation length, but its interval includes zero at ℓ=0.08 m. Adaptive shrinkage therefore separates from unshrunk truncation for the three smoother sampled fields, while the least compatible condition remains inconclusive. Fixed-graph smoothing is distinguishable from GSMA-NF only at ℓ=0.25 m in these pointwise intervals. Together, the mode-energy and error trends link graph-prior benefit to low-mode concentration within the sampled residual family.

### 4.4. Direction-Dependent Transfer and Search Discretization

The transfer-region experiment isolates direction-dependent geometry mismatch through controlled sensor-position perturbations. In each trial, a smooth two-dimensional sensor-position perturbation is drawn from the first seven nonconstant graph modes and rescaled to a root-mean-square magnitude of 0.05 m. The same perturbed geometry generates the anchor and target observations, and all processing uses the nominal coordinates. The direction-independent channel response is set to unity, so the correction estimated at the anchor contains noise and the geometry-induced residual at that reference location. At 2 kHz, target bearing offsets range from $-90^{\circ }$ to $90^{\circ }$ and target ranges span 3–10 m, with 200 paired trials per bearing–range cell.

**Figure 8:**
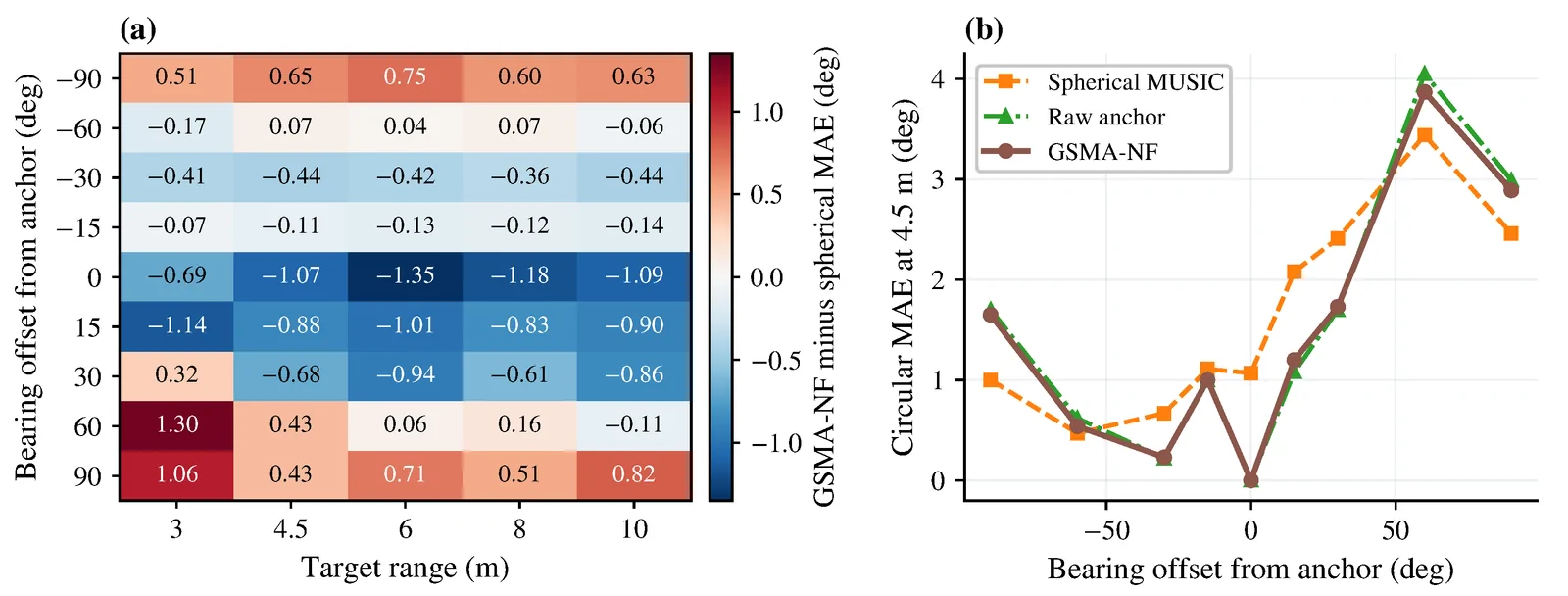
Geometry-induced directional transfer at 0.05 m position-error root mean square: (**a**) paired GSMA-NF-minus-spherical error map and (**b**) method errors at the 4.5 m anchor range.

The paired GSMA-NF-minus-spherical differences in Figure 8a identify a local transfer region. Every sampled cell from $-30^{\circ }$ to $15^{\circ }$ improves with adaptation. At offsets of $\pm 60^{\circ }$, the sign depends on range, and all sampled $\pm 90^{\circ }$ cells favor the nominal spherical model. Across the 45 cells, 27 favor GSMA-NF and 18 favor spherical MUSIC, with paired mean differences from $-1.35^{\circ }$ to $1.30^{\circ }$. Figure 8b shows the asymmetric angular trend at the anchor range, which follows from the irregular nominal geometry and the fixed anchor direction. This controlled result establishes an operating boundary for sensor-position-induced direction dependence under the stated perturbation model. In this experiment, the response oracle uses the unity direction-independent response and therefore coincides with the unadapted spherical model; both methods retain the nominal geometry.

**Figure 9:**
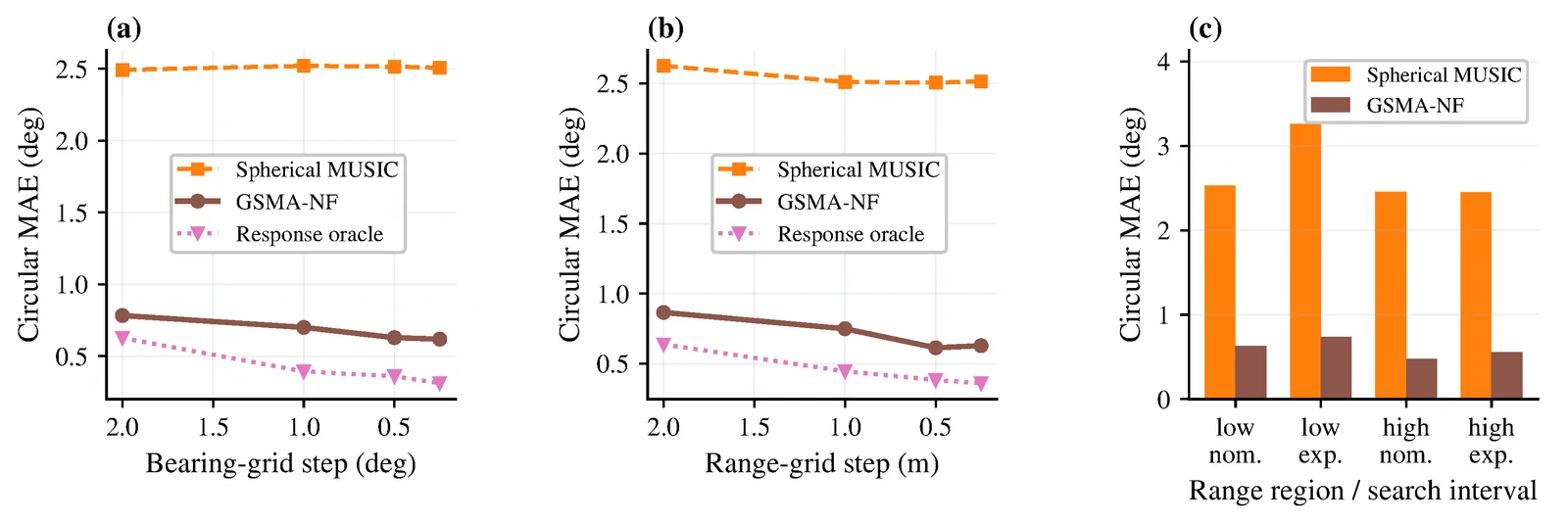
Off-grid search analysis: (**a**) bearing-grid convergence; (**b**) range-grid convergence; (**c**) nominal and expanded range-search intervals near the lower and upper boundaries.

The off-grid study samples true ranges continuously over 3–10 m and reuses 200 paired realizations for every search grid. Across the convergence plots in Figure 9a,b, refining the bearing step from $2^{\circ }$ to $0.25^{\circ }$ changes the GSMA-NF MAE from $0.78^{\circ }$ to $0.62^{\circ }$, while refining the range step from 2 m to 0.25 m changes it from $0.87^{\circ }$ to $0.63^{\circ }$. Spherical MUSIC remains near $2.5^{\circ }$ in both sweeps, so the separation associated with response adaptation persists under finer grids.

The boundary comparison in Figure 9c uses true ranges within 0.25 m of the 3 and 10 m limits. Expanding the search interval from 3–10 m to 2–12 m increases the GSMA-NF MAE from $0.63^{\circ }$ to $0.74^{\circ }$ near the lower boundary and from $0.48^{\circ }$ to $0.55^{\circ }$ near the upper boundary. The nominal interval therefore yields the lower MAE at both sampled boundaries, whereas the expanded candidate set introduces additional competing responses.

### 4.5. Output Screening and Engineering Operating Limits

**Figure 10:**
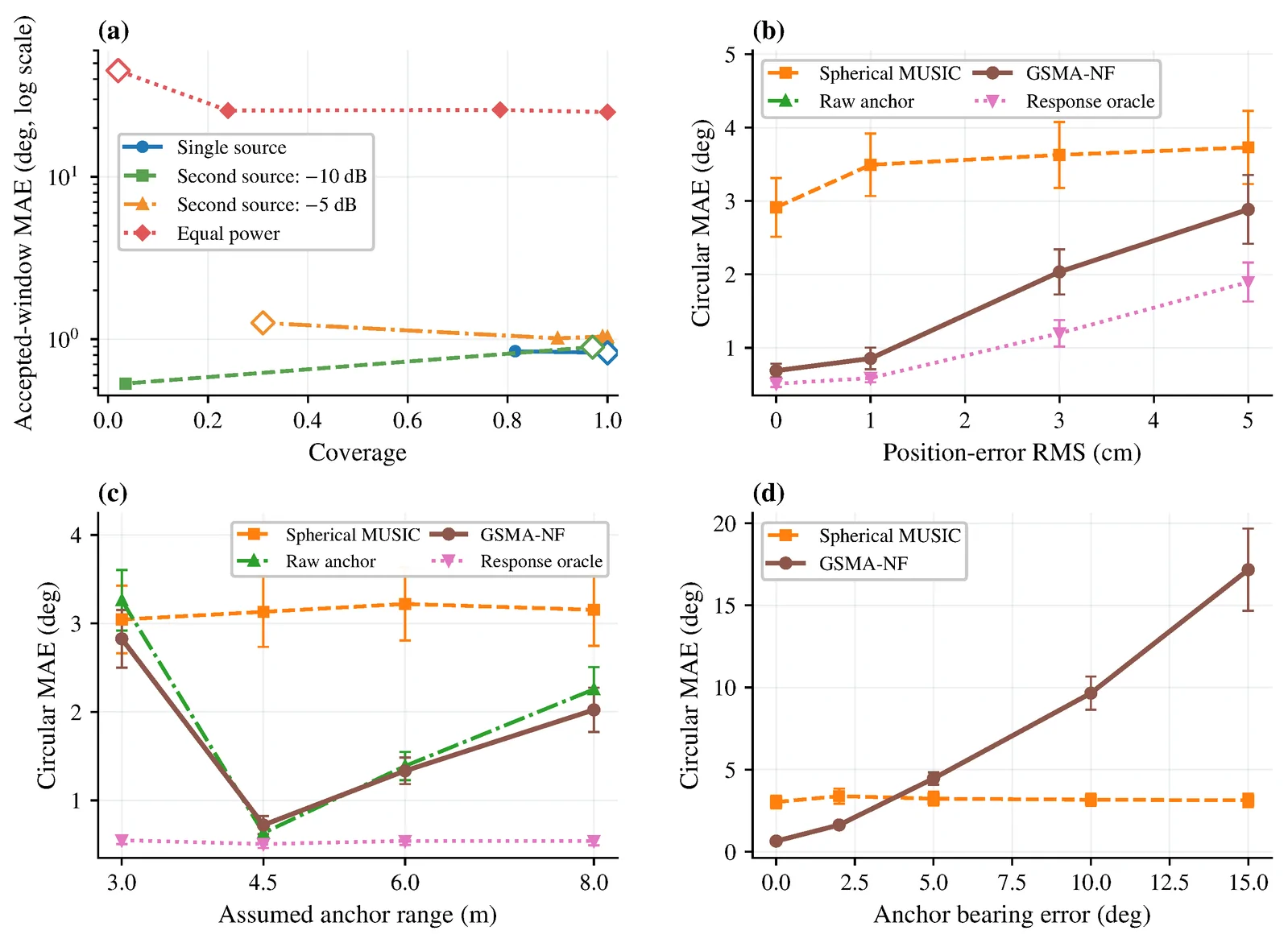
Output-screening and transfer-sensitivity analysis: (**a**) accepted-window mean absolute bearing error versus coverage; (**b**–**d**) bearing error under root-mean-square (RMS) sensor-position error, anchor-range error, and anchor-bearing error.

The coverage–error trajectories in Figure 10a are obtained at 2 kHz, 0 dB target SNR, and 30 snapshots; open diamonds mark $q_{\min}=0.8$. At this operating point, the single-source and weakest-interferer cases retain nearly complete coverage with subdegree accepted-window error. The equal-power case moves to substantially lower coverage and an accepted-window MAE near $1.3^{\circ }$. Together, these trajectories quantify the threshold-dependent relation between output coverage and accepted-window MAE under the tested interference conditions.

The remaining panels identify distinct transfer limits for the anchor-adapted effective response. Figure 10b uses the root-mean-square sensor-position error as the horizontal coordinate. Increasing sensor-position error degrades both GSMA-NF and the response oracle, indicating that direction-dependent geometric perturbations remain after response adaptation at one direction. Anchor-range mismatch is least harmful near the nominal 4.5 m range and becomes more damaging toward the tested extremes. Anchor-bearing error imposes a tighter constraint: GSMA-NF remains below spherical MUSIC at the sampled $2^{\circ }$ error but exceeds it at the sampled $5^{\circ }$ error. These results make reference-bearing accuracy a prerequisite for effective transfer within the sampled operating region.

**Figure 11:**
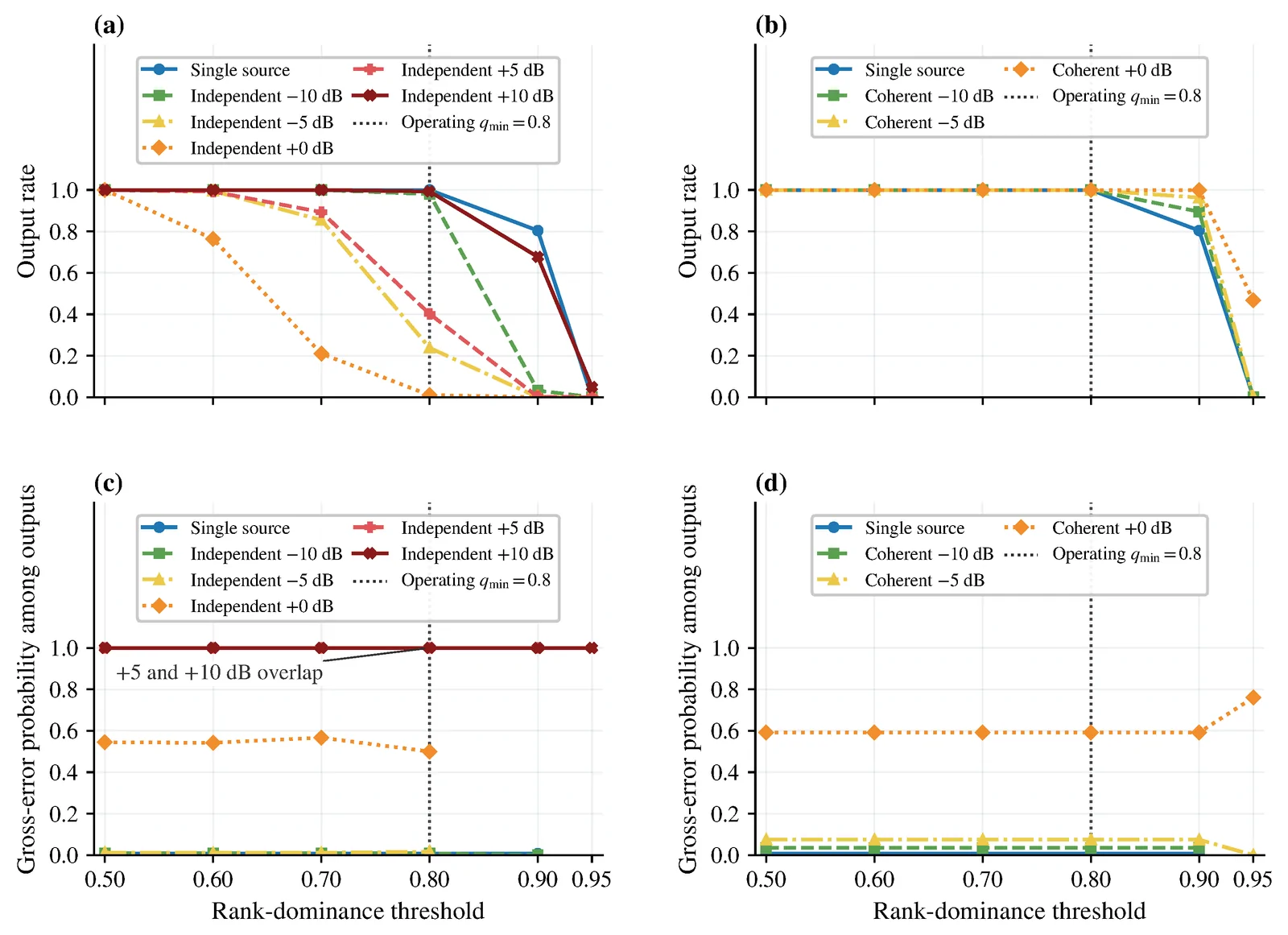
Output-screen stress test: output rates under (**a**) independent and (**b**) coherent interference, and conditional gross-error probabilities under (**c**) independent and (**d**) coherent interference.

The interference stress test uses 1000 paired trials per condition and measures bearing error relative to the designated direct source. An independent nuisance has a random $20^{\circ }$–$80^{\circ }$ separation and its own complex Gaussian waveform, whereas a coherent nuisance shares the direct-source waveform with a random trial-level phase. Both sources are at 5 m, and the interference-to-signal ratio spans −10 to 10 dB. Figure 11a,b trace output rate against the rank-dominance threshold, while Figure 11c,d give the gross-error probability conditional on release. Table 3 reports the accepted counts, Wilson intervals, and accepted-window MAE at $q_{\min}=0.8$; Supplementary Table S4 contains the full threshold scan.

For independent interference, Figure 11a,c show that the screen suppresses most outputs at −5 and 0 dB, yet releases 40.2% and 99.3% at 5 and 10 dB, where every released estimate is a gross error. Under coherent interference, Figure 11b,d retain all outputs at $q_{\min}=0.8$, while the gross-error probability reaches 59.2% at 0 dB. These outcomes establish *q* as a covariance rank-dominance measure and define its function as an output criterion for single-dominant-source model support. Source association and coherent-reflection rejection require additional information.

### 4.6. Fixed-Source Lake-Trial Temporal Compatibility

We analyze a fixed-source lake trial with a custom four-arm array fabricated at Northwestern Polytechnical University (Xi’an, China) from 32 RHC-7 scalar hydrophones (Hangzhou Applied Acoustics Research Institute, Hangzhou, China). A custom 32-channel acquisition unit developed at Northwestern Polytechnical University (Xi’an, China) sampled the scalar signals at 8 kHz. An UW350 projector driven by an SS1000T amplifier (Data Physics Corporation, Riverside, CA, USA) transmitted the narrowband emissions. The receiver and source were deployed at approximate depths of 1 and 5 m, respectively, with an approximately 5 m horizontal separation. Bearing is expressed in the nominal-array frame, whose positive *y* axis points toward the boat stern. The nominal source direction is $226^{\circ }$ and defines the anchor response. The lake trial consequently evaluates temporal transfer and output screening in this coordinate frame, while the controlled experiments quantify bearing accuracy and directional-transfer behavior under known truth.

The field analysis uses coherent, nearly rank-one 2 kHz components in temporally separated intervals. A signal-only inventory selects the 350–365 s anchor interval and the 555–595 s later test sequence from their observed narrowband activity. The former defines manifold adaptation, and the latter evaluates temporal transfer and output screening. Before manifold adaptation, the inventory is formed from 4096-sample Hann frames with a 2048-sample hop and an 8192-point discrete Fourier transform. For each 5 s inventory window, line power is the mean squared magnitude at the 2 kHz bin over sensors and frames. Local noise power is the corresponding median over sensors and the frequency-bin offsets −24 to −8 and 8 to 24, followed by averaging over frames. Their ratio defines the narrowband line SNR in decibels. Candidate activity requires a line SNR of at least 8 dB and the rank-dominance score in Equation (15) to reach 0.80 in at least two consecutive 5 s windows evaluated every 2.5 s. This procedure selects signal-bearing intervals, and the final output screen determines individual reportable estimates for every decision window. Screening depends exclusively on line SNR and rank dominance and is evaluated independently of the nominal anchor bearing and estimated bearing.

**Figure 12:**
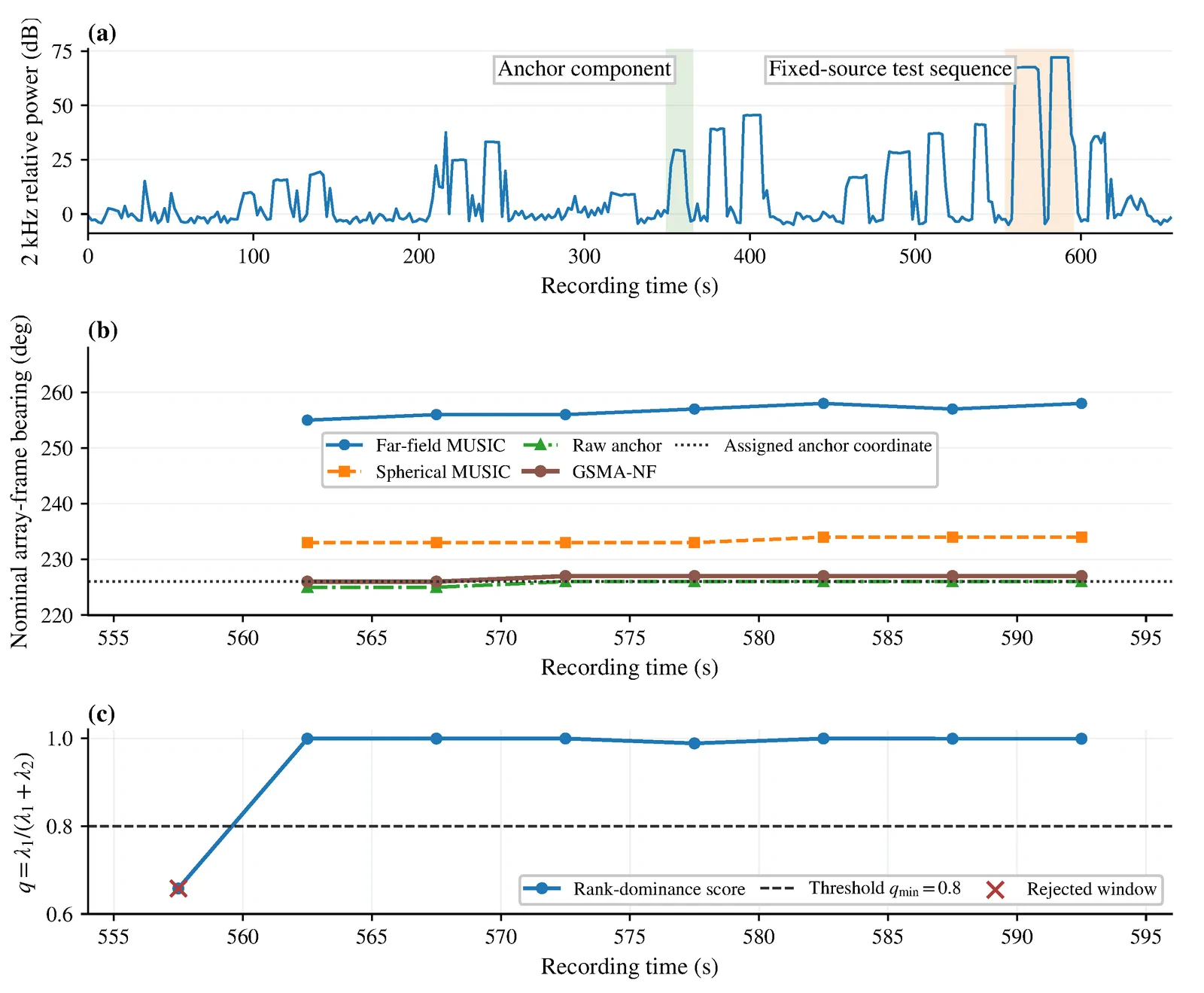
Fixed-source lake-trial analysis of eight decision windows: (**a**) narrowband activity and selected intervals; (**b**) nominal-frame bearing estimates for released windows; (**c**) rank-dominance scores and output decisions.

Anchor adaptation uses the 350–365 s interval, the nominal bearing of $226^{\circ }$, and a nominal horizontal range of 4.5 m; Figure 10c,d evaluates sensitivity to perturbations in the two anchor parameters. The nominal planar coordinates in Figure 2 define the spherical candidates and sensor graph, and processing uses a sound speed of 1500 m/s. The model estimates horizontal bearing in this nominal planar frame. The source–receiver depth difference and other fixed deployment effects beyond this horizontal model contribute to the effective residual inferred at the anchor. Each processed interval is demeaned by channel and divided into 4096-sample Hann-windowed frames with 50% overlap. For each frame, the complex 2 kHz coefficients across the array form one narrowband snapshot. The anchor interval provides 57 snapshots. The later fixed-source test sequence from 555 to 595 s is divided into eight contiguous, nonoverlapping decision windows, each formed from 18 overlapping snapshots. They constitute eight sequential decisions within a single amplitude-sweep realization at one source position. The spherical candidates are evaluated on a $1^{\circ }$ bearing grid and a 0.25 m horizontal-range grid over 3–10 m. For display in Figure 12a, the sensor-averaged 2 kHz coefficient power is evaluated every 2 s and expressed relative to its median over the complete recording.

The activity trace in Figure 12a locates the selected anchor component and later fixed-source sequence within the recording. Figure 12c withholds the 555–560 s onset window and releases the remaining seven windows at $q_{\min}=0.8$. Among those released windows, Figure 12b and Table 4 place the raw-anchor estimates within 225–$226^{\circ }$ and the GSMA-NF estimates within 226–$227^{\circ }$. Together, these descriptive clusters characterize temporal compatibility of the adapted response across the fixed-source sequence. The controlled simulations provide the comparative accuracy and transfer-boundary evidence.

The lake result complements the controlled experiments by demonstrating the complete processing chain on a real fixed-source recording: a measured anchor defines the adapted response, a separately formed test covariance controls output release, and the accepted later windows yield stable local-coordinate bearings. The noisy-anchor simulations in Figure 5 provide the complementary evidence for shrinkage when anchor support is poor, while Figure 8 establishes the geometry-induced transfer boundary.

## 5. Discussion

For the nominal aperture and tested short-range conditions, propagation-model mismatch dominates before response adaptation. The spherical model removes most of the plane-wave bearing error, and GSMA-NF then adapts the remaining effective response. The method addresses an operating setting with nominal geometry, one nominal reference bearing, and a low-dimensional residual prior. Joint gain, phase, and position calibration obtains identifiability from repeated source-relative orientations [10], and signals-of-opportunity geometry calibration accumulates information along a moving trajectory [18]. These approaches solve estimation problems with richer information than the single-anchor setting studied here.

The graph prior is informative when the transferable residual is concentrated in retained low-order modes of the physical sensor graph. Its advantage diminishes as this concentration weakens. The matched generator isolates shrinkage behavior under this operational assumption, and the Gaussian-process study evaluates residuals generated outside that basis. High-order energy serves as an empirical transfer-control proxy in the shrinkage rule. Fixed smoothing remains competitive in several anchor conditions. Comparisons with randomized topologies or alternative geometry-based bases would further isolate the contribution of the selected graph construction.

The directional-transfer map gives a concrete limit for a single anchor under geometry-induced mismatch. Adaptation is beneficial near the reference direction but can degrade bearing estimates at large angular offsets. The reported operating boundary is specific to the tested array geometry, frequency, residual mechanism, and reference quality. The off-grid study shows that the main method separation remains under refined bearing and range grids, with lower boundary-case MAE for the nominal range interval than for the expanded interval.

The rank-dominance screen measures compatibility with a single-dominant-source covariance model. A stronger independent interferer can become the dominant source, and coherent interference can preserve an approximately rank-one covariance. Both mechanisms can yield high *q* and erroneous bearings. Therefore, its evidential role is model-support screening, while source association, source counting, and correctness certification require additional information. In the field data, the estimated correction can combine electronic response, unmodeled elevation, local propagation, and geometry mismatch into one effective residual. The lake experiment shows that this effective response can support stable processing across temporally separated components from one fixed source in the nominal array coordinate system. Controlled simulations provide the truth-referenced accuracy and transfer-boundary evidence. Surveyed multi-bearing field trials would extend the present evidence to absolute field accuracy.

## 6. Conclusions

This paper presented GSMA-NF, a single-anchor manifold-adaptation method for near-field bearing estimation with irregular hydrophone arrays. The method separates nominal spherical propagation from an effective residual response and transfers evidence-supported graph-spectral components. Simulations show that spherical modeling removes most short-range plane-wave error and that shrinkage limits severe negative transfer from weak anchor observations. The graph contribution depends on low-mode concentration, and fixed smoothing remains competitive in some regimes. Geometry-induced directional-transfer tests identify degradation at large offsets from the anchor, while off-grid tests show stable method separation under search-grid refinement. Interference tests establish rank dominance as a model-support check and delineate conditions in which dominant-source structure and bearing correctness diverge. In the fixed-source lake trial, seven released windows demonstrate stable nominal-frame processing across temporally separated narrowband components and illustrate the output screen on measured data. Together, the controlled and field evidence supports GSMA-NF for nominal geometry, a nominal reference bearing, and locally transferable residual structure. Surveyed multi-bearing measurements would extend the present evidence to absolute field accuracy and transfer across source directions.

## Figures and Tables

**Table 1:** Sensitivity of mean absolute circular bearing error to graph bandwidth and graph-mode cutoff.

*h* (m)	K=4	K=6	K=8	K=10	K=12
0.45	1.88	1.29	0.76	0.68	0.63
0.55	1.98	1.32	0.69	0.66	0.63
0.65	1.97	1.19	0.68	0.65	0.65

**Table 2:** Paired mean circular bearing-error differences relative to GSMA-NF under Gaussian-process residual mismatch.

*ℓ* (m)	Low-Order Projection − GSMA-NF	Fixed Graph − GSMA-NF
0.08	1.22 [−0.54, 2.48]	−0.39 [−2.32, 1.39]
0.25	1.44 [0.88, 2.11]	0.80 [0.21, 1.56]
0.55	0.57 [0.17, 1.02]	−0.06 [−0.30, 0.17]
1.10	0.74 [0.10, 1.52]	0.14 [−0.29, 0.65]

**Table 3:** Output-screen performance at $q_{\min}=0.8$ in 1000 trials per condition, including a 95% confidence interval (CI) for each reported rate.

Condition	Accepted	Output Rate (95% CI)	Gross-Error Rate (95% CI)	Accepted MAE (^◦^)
Single source	1000	1.000 (0.996, 1.000)	0.007 (0.003, 0.014)	0.68
Independent, −10 dB	980	0.980 (0.969, 0.987)	0.009 (0.005, 0.017)	0.72
Independent, −5 dB	238	0.238 (0.213, 0.265)	0.017 (0.007, 0.042)	0.80
Independent, 0 dB	10	0.010 (0.005, 0.018)	0.500 (0.237, 0.763)	20.27
Independent, 5 dB	402	0.402 (0.372, 0.433)	1.000 (0.991, 1.000)	48.37
Independent, 10 dB	993	0.993 (0.986, 0.997)	1.000 (0.996, 1.000)	49.04
Coherent, −10 dB	1000	1.000 (0.996, 1.000)	0.035 (0.025, 0.048)	0.97
Coherent, −5 dB	1000	1.000 (0.996, 1.000)	0.075 (0.060, 0.093)	1.47
Coherent, 0 dB	1000	1.000 (0.996, 1.000)	0.592 (0.561, 0.622)	26.34

**Table 4:** Nominal-frame bearing estimates across the released lake-trial windows.

Method	Released Windows	Mean (^◦^)	Observed Range (^◦^)
Far-field MUSIC	7/8	256.71	255–258
Spherical MUSIC	7/8	233.43	233–234
Raw anchor	7/8	225.71	225–226
GSMA-NF	7/8	226.71	226–227

## Data Availability

The data and materials supporting this study are available from the first author upon reasonable request.

## Supplementary Materials

The following supporting information can be downloaded at https://www.mdpi.com/article/10.3390/s26216811/s1, Table S1: Nominal scalar-hydrophone coordinates used in the simulations and lake-data processing; Table S2: Protocols for the added numerical studies, including SNR and snapshot settings; Table S3: Repeated-subsample stability of weak-anchor 95th-percentile error and gross-error rate; Table S4: Output screening across all tested thresholds with Wilson confidence intervals.

## References

[1] Bossér D., Hendeby G., Nordenvaad M.L., Skog I. (2025). Broadband passive sonar track-before-detect using raw acoustic data. IEEE J. Ocean. Eng..

[2] Hou X., Chen Y., Zhang B., Yang Y. (2024). Review of Research Progress on Passive Direction-of-Arrival Tracking Technology for Underwater Targets. Remote Sens..

[3] Antonelli G., Biondi A., Caiti A., Chiaverini S., Costanzi R., Di Lillo P., Gentili A., Munafò A. (2026). Towards a Multi-Robot Architecture for Bearing-Only Tracking of Underwater Acoustic Sources Using Vector Sensors. Ocean Eng..

[4] Etter P.C. (2018). Underwater Acoustic Modeling and Simulation.

[5] Zhang Y., Zhang X., Yang J. (2026). Tensor-Based DOA Estimation for Hybrid Scalar-Vector Sensor Arrays. Front. Mar. Sci..

[6] Schmidt R.O. (1986). Multiple emitter location and signal parameter estimation. IEEE Trans. Antennas Propag..

[7] Stoica P., Nehorai A. (1989). MUSIC, maximum likelihood, and Cramér–Rao bound. IEEE Trans. Acoust. Speech Signal Process..

[8] Molaei A.M., Zakeri B., Andargoli S.M.H., Abbasi M.A.B., Fusco V., Yurduseven O. (2024). A comprehensive review of direction-of-arrival estimation and localization approaches in mixed-field sources scenario. IEEE Access.

[9] Wang F., Jiang R., Tian X., Liu X., Jiang S., Gu B., Chen Y. (2022). An Efficient Near-Field Model Gain-Phase Self-Calibration Method for Uniform Rectangular Arrays. IEEE J. Ocean. Eng..

[10] Zhang Z., Zhou T., Du W., Guo Q. (2025). A Near-Field Gain-Phase and Position Errors Calibration Method for Acoustic Arrays. IEEE Trans. Instrum. Meas..

[11] Stinco P., Tesei A., Been R. (2024). Adaptive correcting combiner for arrays of acoustic vector sensors with phase and gain mismatch errors. Appl. Acoust..

[12] Xu M., Wu J., Liu L. (2025). Two-Dimensional DOA Estimation of Underwater Acoustic Signals with Gain-Phase Errors. IEEE Trans. Commun..

[13] Flanagan B.P., Bell K.L. (2001). Array self-calibration with large sensor position errors. Signal Process..

[14] Sabra K.G., Roux P., Thode A.M., D’Spain G.L., Hodgkiss W.S., Kuperman W.A. (2005). Using ocean ambient noise for array self-localization and self-synchronization. IEEE J. Ocean. Eng..

[15] Liu Y., Liu C., Zhao Y., Zhu J. (2018). Wideband array self-calibration and DOA estimation under large position errors. Digit. Signal Process..

[16] Ramamohan K.N., Chepuri S.P., Fernandez Comesana D., Leus G. (2023). Self-calibration of acoustic scalar and vector sensor arrays. IEEE Trans. Signal Process..

[17] Zhang W., Jiang P., Lin J., Sun J. (2024). Array Shape Calibration Based on Coherence of Noise Radiated by Non-Cooperative Ships. Ocean Eng..

[18] Skog I., Nordenvaad M.L., Hendeby G. (2024). Signals-of-opportunity-based hydrophone array shape and orientation estimation. IEEE J. Ocean. Eng..

[19] Wang J., Pan X., Li A., Liu F., Jiao J. (2024). Fast Joint Estimation of Direction of Arrival and Towed Array Shape Based on Marginal Likelihood Maximization. Digit. Signal Process..

[20] Zhang X., Yan Z., Li X., Li Z., Tang J., Luo Z. (2026). Self-calibrated SBL for joint array deformation and DOA estimation in underwater acoustic vertical linear arrays. Ocean Eng..

[21] Bai Z., Liu C., Zhang J., Wang J. (2025). Enhancing robust acoustic DOA estimation against position errors via fast sparse Bayesian learning. Appl. Soft Comput..

[22] Du Z., Hao Y., Qiu L., Li C., Liang G. (2024). Sparsity-Based Direction-of-Arrival Estimation in the Presence of Near-Field and Far-Field Interferences for Small-Scale Platform Sonar Arrays. J. Acoust. Soc. Am..

[23] Zhang M., Zhu Z.D. (1995). Direction finding with unknown array sensor gains and phases. Int. J. Electron..

[24] Dai Z., Su W., Gu H. (2020). A Calibration Method for Linear Arrays in the Presence of Gain-Phase Errors. IEICE Trans. Fundam. Electron. Commun. Comput. Sci..

[25] Liu C., Tang X., Zhang Z. (2023). A New Gain-Phase Error Pre-Calibration Method for Uniform Linear Arrays. Sensors.

[26] Moreira L.A.S., Ramos A.L.L., de Campos M.L.R., Apolinário J.A., Serrenho F.G. (2019). A Graph Signal Processing Approach to Direction of Arrival Estimation. Proceedings of the 2019 27th European Signal Processing Conference (EUSIPCO).

[27] Alcantara E., Atlas L., Abadi S. (2021). Direction-of-Arrival Estimation Using Signal Processing on Graphs. Proceedings of the 2021 IEEE Statistical Signal Processing Workshop (SSP).

[28] Li D., Liu W., Zakharov Y., Mitchell P.D. (2023). Graph Signal Processing for Narrowband Direction of Arrival Estimation. Proceedings of the ICASSP 2023–2023 IEEE International Conference on Acoustics, Speech and Signal Processing.

[29] Shen J., Ying T., Zhang Z., Wei Y., Liu W., Shi Z. (2024). Direction-of-Arrival Estimation with Efficient Graph Spatial Spectrum Construction. Proceedings of the 2024 IEEE/CIC International Conference on Communications in China Workshops.

[30] Liao K., Yu Z., Xie N., Jiang J. (2022). Joint Estimation of Azimuth and Distance for Far-Field Multi Targets Based on Graph Signal Processing. Remote Sens..

[31] Liu K., Zou C., Ma J. (2025). Motion Coprime Array-Based DOA Estimation Considering Phase Disturbance of Sensor Array. Appl. Acoust..

[32] Zhang Q., Wang H., Shen X., Yan Y., Zhu Y., Jensen J.R. (2025). Enhancing Underwater Single Snapshot DOA Estimation for Limited Dataset with Modified Knowledge Distillation. Appl. Acoust..

[33] Tao Q., Fan J., Qian Z., Fu X. (2025). Temporal-Spatial Two-Dimensional Sparse Deconvolution Beamforming for Wideband Underwater Acoustic Multipath Signals. Measurement.

[34] Khurjekar I.D., Gerstoft P. (2023). Uncertainty Quantification for Direction-of-Arrival Estimation with Conformal Prediction. J. Acoust. Soc. Am..

[35] Hou X., Qiao Y., Zhang B., Yang Y. (2023). Robust Underwater Direction-of-Arrival Tracking Based on AI-Aided Variational Bayesian Extended Kalman Filter. Remote Sens..

[36] Shuman D.I., Narang S.K., Frossard P., Ortega A., Vandergheynst P. (2013). The Emerging Field of Signal Processing on Graphs: Extending High-Dimensional Data Analysis to Networks and Other Irregular Domains. IEEE Signal Process. Mag..

[37] Ortega A., Frossard P., Kovačević J., Moura J.M.F., Vandergheynst P. (2018). Graph Signal Processing: Overview, Challenges, and Applications. Proc. IEEE.

