# Supplementary material for: Single-Anchor Graph-Spectral Manifold Adaptation for Near-Field Underwater Bearing Estimation
Source: Sensors (Basel). 2026 Oct 28;26(21):6811. doi: 10.3390/s26216811 (PMC13766145; doi:10.3390/s26216811)
Supplement: Supplementary file 1 [file sensors-26-06811-s001.zip › sensors-4589350-supplementary.pdf]

Supplementary Information

Single-Anchor Graph-Spectral Manifold Adaptation for Near-Field Underwater Bearing Estimation

Yi Zhang, Xiaomin Zhang, and Jie Yang

This document reports the nominal scalar-hydrophone coordinates and extended numerical results for graph-spectral manifold adaptation for near-field bearing estimation (GSMA-NF). The central vector sensor shown in the deployment records is not used by the 32-channel scalar-array processing. Supplementary Tables S1–S4 provide the geometry, experiment protocols, field-window audit, and complete screening-threshold results referenced in the revised manuscript.

Nominal Array Geometry

Table S1: Nominal scalar-hydrophone coordinates used in the simulations and lake-data processing. Coordinates are relative to the array-coordinate origin; an independent metrological accuracy record is unavailable.

Sensor	Support arm	x (m)	y (m)
1	1	0.277128	0.000000
2	1	0.493634	−0.125000
3	1	0.710141	−0.250000
4	1	0.926647	−0.375000
5	1	1.143154	−0.500000
6	1	1.359660	−0.625000
7	1	1.576166	−0.750000
8	1	1.792673	−0.875000
9	1	2.009179	−1.000000
10	1	2.225685	−1.125000
11	2	−0.277128	0.000000
12	2	−0.493634	−0.125000
13	2	−0.710141	−0.250000
14	2	−0.926647	−0.375000
15	2	−1.143154	−0.500000
16	2	−1.359660	−0.625000
17	2	−1.576166	−0.750000
18	2	−1.792673	−0.875000
19	2	−2.009179	−1.000000
20	2	−2.225685	−1.125000
21	3	0.360000	−0.307846
22	3	0.360000	−0.557846
23	3	0.360000	−0.807846
24	3	0.360000	−1.057846
25	3	0.360000	−1.307846
26	3	0.360000	−1.557846
27	4	−0.360000	−0.307846
28	4	−0.360000	−0.557846
29	4	−0.360000	−0.807846
30	4	−0.360000	−1.057846
31	4	−0.360000	−1.307846

Table S1 continued.

Sensor	Support arm	x (m)	y (m)
32	4	−0.360000	−1.557846

Revision Experiment Protocols

All methods compared within a condition use paired source, response, bearing, and noise realizations. Each experiment group uses a distinct fixed pseudorandom stream. Signal and noise coefficients are zero-mean circular complex Gaussian variables unless noted otherwise.

Table S2: Protocols for the added numerical studies, including signal-to-noise ratio (SNR) and snapshot settings.

Study	Conditions
Anchor quality	Anchor SNR $\{-10, -5, 0, 10\}$ dB; anchor snapshots $\{8, 20, 50\}$; 500 trials per cell
Weak-anchor tail	Anchor SNR -10 dB; 8 anchor snapshots; 2000 paired trials
Target support	Target SNR $\{-10, -5, 0, 5, 10\}$ dB; target snapshots $\{8, 18, 30, 64, 128, 256\}$; 300 trials per cell
Directional transfer	Bearing offsets $\{-90, -60, -30, -15, 0, 15, 30, 60, 90\}°$; ranges $\{3, 4.5, 6, 8, 10\}$ m; 0.05 m root-mean-square position error; 200 trials per cell
Search discretization	Continuous true ranges; bearing steps $\{2, 1, 0.5, 0.25\}°$; range steps $\{2, 1, 0.5, 0.25\}$ m; 200 paired trials
Screening stress	Independent interferer levels $\{-10, -5, 0, 5, 10\}$ dB; coherent levels $\{-10, -5, 0\}$ dB; 1000 trials per condition

For the directional-transfer study, a two-dimensional position perturbation is drawn from the first seven nonconstant graph modes and rescaled to 0.05 m root-mean-square magnitude. The same perturbed geometry generates the anchor and target data in a trial; all estimators use the nominal geometry. Channel response is unity. This construction isolates direction-dependent residuals created by geometry error and does not model lake multipath.

For the screening study, the direct and nuisance sources are both at 5 m. The nuisance bearing separation is uniform over 20°–80° with random sign. An independent nuisance has its own complex Gaussian source sequence. A coherent nuisance shares the direct-source sequence with one random trial-level relative phase. Bearing error is measured relative to the direct source.

Lake-Trial Window Definition

The deployment record identifies a fixed-source test with an approximately 1 m receiver depth, an approximately 5 m source depth, an approximately 5 m horizontal separation, and a sequence of transmitted frequencies. The field analysis uses observed coherent 2 kHz activity in two temporally separated intervals: the 350–365 s anchor interval defines manifold adaptation, and the 555–595 s test interval evaluates temporal transfer and output screening.

The processing record assigns the anchor a local bearing of 226°. This assignment predates GSMA-NF and is external to the proposed adaptation, but it is not an independently surveyed field bearing. The deployment record identifies the positive y direction of the nominal array coordinates as pointing toward the boat stern but does not provide an independently surveyed array-frame bearing for the analyzed sequence. The lake result is therefore interpreted in the recorded local coordinate system and not as an absolute field-accuracy test.

The field-window audit summarized here records the eight contiguous 5 s decision windows from 555 to 595 s, their rank-dominance scores, and their release decisions at $q_{\min} = 0.8$. The 555–560 s onset window is the only rejected window. Each window contains 18 snapshots under the documented 8 kHz sampling rate, 4096-sample frame, and 2048-sample hop. These windows sample one fixed-source amplitude-sweep sequence and do not constitute independent field replicates.

Other archived field recordings use a different sensor configuration and do not provide matched scalar-array measurements with independently surveyed array-frame bearings. They are therefore not combined with this analysis or used to claim multi-bearing field validation.

Heavy-Tail and Sample-Size Results

Table S3: Repeated-subsample stability of the 95th-percentile error (P95) and gross-error rate for the weak-anchor condition; brackets give the central 90% range over 200 repeated subsamples, except at the complete sample size.

N	Raw P95 (°)	GSMA-NF P95 (°)	Raw gross rate	GSMA-NF gross rate
200	74.46 [42.64, 112.72]	9.18 [7.97, 10.65]	0.350 [0.300, 0.400]	0.180 [0.140, 0.220]
500	74.59 [57.52, 96.46]	9.24 [8.46, 10.40]	0.354 [0.322, 0.380]	0.184 [0.162, 0.210]
1000	78.36 [66.52, 93.78]	9.26 [8.72, 9.90]	0.355 [0.338, 0.371]	0.186 [0.174, 0.199]
2000	77.27	9.33	0.354	0.185

Complete Screening-Threshold Sweep

The gross-error threshold is 5°. For each screening threshold, Table S4 reports the accepted count and output rate among 1000 trials, together with the gross-error probability conditional on output release. Both rates include 95% Wilson confidence intervals. When no estimate is released, the conditional gross-error probability and its interval are undefined (shown as dashes).

Table S4: Output screening across all tested thresholds (1000 trials per condition). Each reported rate is followed by its 95% Wilson confidence interval.

Condition	$q_{\min}$	Accepted	Output rate (95% CI)	Gross-error rate (95% CI)
Single source	0.50	1000	1.000 (0.996, 1.000)	0.007 (0.003, 0.014)
Single source	0.60	1000	1.000 (0.996, 1.000)	0.007 (0.003, 0.014)
Single source	0.70	1000	1.000 (0.996, 1.000)	0.007 (0.003, 0.014)
Single source	0.80	1000	1.000 (0.996, 1.000)	0.007 (0.003, 0.014)
Single source	0.90	804	0.804 (0.778, 0.827)	0.007 (0.003, 0.016)
Single source	0.95	0	0.000 (0.000, 0.004)	–
Independent, −10 dB	0.50	1000	1.000 (0.996, 1.000)	0.009 (0.005, 0.017)
Independent, −10 dB	0.60	1000	1.000 (0.996, 1.000)	0.009 (0.005, 0.017)
Independent, −10 dB	0.70	1000	1.000 (0.996, 1.000)	0.009 (0.005, 0.017)
Independent, −10 dB	0.80	980	0.980 (0.969, 0.987)	0.009 (0.005, 0.017)
Independent, −10 dB	0.90	32	0.032 (0.023, 0.045)	0.000 (0.000, 0.107)
Independent, −10 dB	0.95	0	0.000 (0.000, 0.004)	–
Independent, −5 dB	0.50	1000	1.000 (0.996, 1.000)	0.012 (0.007, 0.021)
Independent, −5 dB	0.60	995	0.995 (0.988, 0.998)	0.011 (0.006, 0.020)
Independent, −5 dB	0.70	855	0.855 (0.832, 0.875)	0.012 (0.006, 0.021)
Independent, −5 dB	0.80	238	0.238 (0.213, 0.265)	0.017 (0.007, 0.042)
Independent, −5 dB	0.90	0	0.000 (0.000, 0.004)	–
Independent, −5 dB	0.95	0	0.000 (0.000, 0.004)	–
Independent, 0 dB	0.50	1000	1.000 (0.996, 1.000)	0.545 (0.514, 0.576)
Independent, 0 dB	0.60	764	0.764 (0.737, 0.789)	0.542 (0.506, 0.577)
Independent, 0 dB	0.70	210	0.210 (0.186, 0.236)	0.567 (0.499, 0.632)
Independent, 0 dB	0.80	10	0.010 (0.005, 0.018)	0.500 (0.237, 0.763)
Independent, 0 dB	0.90	0	0.000 (0.000, 0.004)	–
Independent, 0 dB	0.95	0	0.000 (0.000, 0.004)	–
Independent, 5 dB	0.50	1000	1.000 (0.996, 1.000)	1.000 (0.996, 1.000)
Independent, 5 dB	0.60	992	0.992 (0.984, 0.996)	1.000 (0.996, 1.000)
Independent, 5 dB	0.70	893	0.893 (0.872, 0.911)	1.000 (0.996, 1.000)
Independent, 5 dB	0.80	402	0.402 (0.372, 0.433)	1.000 (0.991, 1.000)
Independent, 5 dB	0.90	2	0.002 (0.001, 0.007)	1.000 (0.342, 1.000)

Table S4 continued.

Condition	$q_{\min}$	Accepted	Output rate (95% CI)	Gross-error rate (95% CI)
Independent, 5 dB	0.95	0	0.000 (0.000, 0.004)	–
Independent, 10 dB	0.50	1000	1.000 (0.996, 1.000)	1.000 (0.996, 1.000)
Independent, 10 dB	0.60	1000	1.000 (0.996, 1.000)	1.000 (0.996, 1.000)
Independent, 10 dB	0.70	1000	1.000 (0.996, 1.000)	1.000 (0.996, 1.000)
Independent, 10 dB	0.80	993	0.993 (0.986, 0.997)	1.000 (0.996, 1.000)
Independent, 10 dB	0.90	677	0.677 (0.647, 0.705)	1.000 (0.994, 1.000)
Independent, 10 dB	0.95	49	0.049 (0.037, 0.064)	1.000 (0.927, 1.000)
Coherent, −10 dB	0.50	1000	1.000 (0.996, 1.000)	0.035 (0.025, 0.048)
Coherent, −10 dB	0.60	1000	1.000 (0.996, 1.000)	0.035 (0.025, 0.048)
Coherent, −10 dB	0.70	1000	1.000 (0.996, 1.000)	0.035 (0.025, 0.048)
Coherent, −10 dB	0.80	1000	1.000 (0.996, 1.000)	0.035 (0.025, 0.048)
Coherent, −10 dB	0.90	895	0.895 (0.874, 0.913)	0.036 (0.025, 0.050)
Coherent, −10 dB	0.95	0	0.000 (0.000, 0.004)	–
Coherent, −5 dB	0.50	1000	1.000 (0.996, 1.000)	0.075 (0.060, 0.093)
Coherent, −5 dB	0.60	1000	1.000 (0.996, 1.000)	0.075 (0.060, 0.093)
Coherent, −5 dB	0.70	1000	1.000 (0.996, 1.000)	0.075 (0.060, 0.093)
Coherent, −5 dB	0.80	1000	1.000 (0.996, 1.000)	0.075 (0.060, 0.093)
Coherent, −5 dB	0.90	963	0.963 (0.949, 0.973)	0.075 (0.060, 0.093)
Coherent, −5 dB	0.95	3	0.003 (0.001, 0.009)	0.000 (0.000, 0.561)
Coherent, 0 dB	0.50	1000	1.000 (0.996, 1.000)	0.592 (0.561, 0.622)
Coherent, 0 dB	0.60	1000	1.000 (0.996, 1.000)	0.592 (0.561, 0.622)
Coherent, 0 dB	0.70	1000	1.000 (0.996, 1.000)	0.592 (0.561, 0.622)
Coherent, 0 dB	0.80	1000	1.000 (0.996, 1.000)	0.592 (0.561, 0.622)
Coherent, 0 dB	0.90	999	0.999 (0.994, 1.000)	0.592 (0.561, 0.622)
Coherent, 0 dB	0.95	468	0.468 (0.437, 0.499)	0.761 (0.720, 0.797)
